# Re-evaluation of the WHO (2010) formaldehyde indoor air quality guideline for cancer risk assessment

**DOI:** 10.1007/s00204-016-1733-8

**Published:** 2016-05-21

**Authors:** Gunnar Damgård Nielsen, Søren Thor Larsen, Peder Wolkoff

**Affiliations:** National Research Centre for the Working Environment, Lersø Parkallé 105, 2100 Copenhagen, Denmark

**Keywords:** Formaldehyde, World Health Organization, Indoor air guideline, Cancer, Risk assessment

## Abstract

In 2010, the World Health Organization (WHO) established an indoor air quality guideline for short- and long-term exposures to formaldehyde (FA) of 0.1 mg/m^3^ (0.08 ppm) for all 30-min periods at lifelong exposure. This guideline was supported by studies from 2010 to 2013. Since 2013, new key studies have been published and key cancer cohorts have been updated, which we have evaluated and compared with the WHO guideline. FA is genotoxic, causing DNA adduct formation, and has a clastogenic effect; exposure–response relationships were nonlinear. Relevant genetic polymorphisms were not identified. Normal indoor air FA concentrations do not pass beyond the respiratory epithelium, and therefore FA’s direct effects are limited to portal-of-entry effects. However, systemic effects have been observed in rats and mice, which may be due to secondary effects as airway inflammation and (sensory) irritation of eyes and the upper airways, which *inter alia* decreases respiratory ventilation. Both secondary effects are prevented at the guideline level. Nasopharyngeal cancer and leukaemia were observed inconsistently among studies; new updates of the US National Cancer Institute (NCI) cohort confirmed that the relative risk was not increased with mean FA exposures below 1 ppm and peak exposures below 4 ppm. Hodgkin’s lymphoma, not observed in the other studies reviewed and not considered FA dependent, was increased in the NCI cohort at a mean concentration ≥0.6 mg/m^3^ and at peak exposures ≥2.5 mg/m^3^; both levels are above the WHO guideline. Overall, the credibility of the WHO guideline has not been challenged by new studies.

## Introduction

Formaldehyde (FA; 1 ppm = 1.23 mg/m^3^ at 1 atm and 25 °C) is a high-volume chemical, which is used for disinfection purposes and as a preservative. Also, it is used in the production of resins and binders, which are used in wood-products (e.g. particle board and plywood), pulp and paper, and mineral wool. Furthermore, FA is used in the production of plastics, coatings and paints, flooring materials, for textile finishing, for synthesis of chemicals, and it is a component of combustion products (Salthammer et al. 2010; IARC 2012). Additionally, FA is a major compound derived from ozone-initiated reactions with alkenes, e.g. terpenes (Atkinson and Arey 2003). Due to its ubiquitous use, FA is a common indoor air pollutant.

The majority of the studies showed that indoor air concentrations in Europe and the USA were below 100 µg/m^3^ and the median, geometric mean or arithmetic means ranged between 5 and 60 µg/m^3^ (Salthammer et al. 2010; Sarigiannis et al. 2011). Overall, these levels are supported by recent studies in industrialized countries. Thus, nursing homes for elderly people in seven countries in Europa had a mean (8 h) FA concentration of 7 µg/m^3^ and a maximum concentration of 21 µg/m^3^ (Bentayeb et al. 2015). In a study in French dwellings, the range of FA concentrations varied from 18 to 26 µg/m^3^ (Brown et al. 2015). In another study in French dwellings, the mean, 90th percentile and the maximum concentration was 29, 46 and 113 µg/m^3^, respectively, in children’s bedrooms (Dallongeville et al. 2015). Another study compared apartments in Finland and in Lithuania (Du et al. 2015). The mean and maximum concentrations were 17.5 and 40 µg/m^3^ and 23 and 51 µg/m^3^, respectively. A Spanish study showed that homes in a Spanish city had a mean, 75th percentile and maximum FA concentration of 55, 74 and 91 µg/m^3^, respectively (Villanueva et al. 2015). In another Spanish study, no difference was found in FA concentrations in indoor air concentrations in the bedrooms, living rooms and non-industrial workplaces, mainly offices, where the mean concentration was about 25 µg/m^3^ and ranged from 6 to 48 µg/m^3^ (Rovira et al. 2016). Mullen et al. (2016) showed that in Californian homes, the 25th and 75th percentiles were 12 and 25 µg/m^3^, respectively, with a maximum of 50 µg/m^3^. In another Californian study, the mean FA concentration was 34 µg/m^3^ in homes built with low-emitting materials and 46 µg/m^3^ in conventional homes at an air exchange rate of 0.35 h^−1^ (Hult et al. 2015). Furthermore, 40 early childhood education facilities were studied in California; the arithmetic mean FA concentration was 19 µg/m^3^ with a range from 0.7 to 49 µg/m^3^ (Bradman et al. 2016). In houses inhabited by asthmatics in the Boston area, the geometric mean FA concentration was 43 µg/m^3^. The concentrations ranged from 6 to 162 µg/m^3^, and 6 % of the houses had a FA concentration exceeding 122 µg/m^3^ (Dannemiller et al. 2013). In homes in Australia, the mean and maximum FA concentration was 15 and 46 µg/m^3^, respectively (Lazenby et al. 2012). In homes in Japan, the mean and maximum concentration was 13 and 58 µg/m^3^, respectively, in the winter and in the summer 34 and 220 µg/m^3^, respectively, with 0.7 % exceeding 100 µg/m^3^ (Uchiyama et al. 2015). In Korea, in newly built apartments at the pre-occupancy stage, the mean, the 95th percentile and the maximum FA concentration was 61, 110 and 160 µg/m^3^, respectively (Shin and Jo 2012). In apartments in a Chinese city, the mean (range) concentration was 100 (80–130) µg/m^3^ in living rooms (Zhu and Liu 2014). In Beijing, even higher concentrations were found in dwellings and offices that had been remodelled within the past year. Thus, the mean (±SD) was 131 ± 90 µg/m^3^ in dwellings with a maximum concentration of 800 and 85 ± 56 µg/m^3^ in offices with a maximum concentration of 300 µg/m^3^ (Huang et al. 2013).

Many countries have set guideline values for indoor air FA (Salthammer et al. 2010). The World Health Organization (2010) set an indoor air quality guideline (IAQG) for FA at 0.1 mg/m^3^ (0.08 ppm), which applies to all 30-min periods lifelong. The guideline was further supported by extended literature reviews (Nielsen and Wolkoff 2010; Wolkoff and Nielsen 2010). Shortly after the WHO presented its recommendation, Golden (2011) also analysed the FA data and proposed an indoor air guideline value of 0.1 ppm (0.12 mg/m^3^). A recent update by IARC (2012) classified FA as “carcinogenic to humans (Group 1)” on the basis that FA may cause cancer of the nasopharynx and leukaemia, whereas there was limited evidence for association with sinonasal cancer. However, a consistent finding is the observed occurrence of nasal cancer in rats and mice at high FA exposures.

Many new key studies have been published that have been used in this re-evaluation of the WHO (2010) IAQG. Previous conclusions have been summarized from the evaluations (WHO 2010; Nielsen and Wolkoff 2010; Wolkoff and Nielsen 2010; Golden 2011; RAC 2012; Nielsen et al. 2013; NRC 2014). The focus of this review is recent studies (mainly years ≥ 2013); however, for transparency reasons, earlier key studies have also been included when considered appropriate. We have excluded new occupational studies that do not include measured FA concentrations, studies where the exposure–response relationships could not be evaluated (e.g. Attia et al. 2014; Santovito et al. 2014), and studies with complex environmental exposures (e.g. Vilavert et al. 2014) where no measured health effect was included or where a low-level environmental FA exposure was a proxy of an exposure to a complex outdoor air mixture (e.g. Marcon et al. 2014) as such exposures do not allow disentangling of the effects of FA. It should be noted that this does not mean that such studies are not useful for risk management purposes. Also, we excluded animal studies with mixtures where effects of FA could not be disentangled (e.g. Wang et al. 2013a) and studies with exposures to FA aerosols (e.g. Lima et al. 2015) as the WHO IAQG is set for gaseous FA. Although the specific purpose is the evaluation of the WHO IAQG for FA, the evaluated studies are also relevant for setting other guidelines or standards for FA, for example, occupational exposure limits.

## Absorption, distribution, metabolism and elimination

Due to its high water solubility and reactivity, airborne FA is absorbed mainly (~90 %) in the upper airways (c.f. WHO 2010; Nielsen et al. 2013). In the aqueous tissue phase, FA adds water, forming methandiol (methylene glycol, CH_2_(OH)_2_), accounting for more than 99.9 % of total FA in the aqueous phase. CH_2_(OH)_2_ is in equilibrium with free FA (<0.1 %) in the water phase, where CH_2_(OH)_2_ serves as a FA liberator. CH_2_(OH)_2_ itself may have a low toxicity (Golden and Valantini 2014). In the tissue, FA forms adducts and cross-links with RNA, DNA and proteins, including DNA–protein cross-links (DPX). In rat nasal tissue, DPX increases disproportionately at exposure levels above 2–3 ppm. FA is an endogenous metabolite, and its blood concentration is about 2–3 mg FA/L. The half-life of FA in blood is about 1–1.5 min. FA is metabolized to formate, which is incorporated in tissue components via the one-carbon pool, excreted in the urine or oxidized to carbon dioxide (c.f. WHO 2010; Nielsen et al. 2013). Using a specific (unbiased) method, the FA concentration in air exhaled through the mouth was found at levels up to 1.7 ppb; this figure may be higher shortly after smoking a cigarette. However, the concentration was below 0.5 ppb in most cases (Riess et al. 2010). The exhaled FA concentration may be higher in air breathed through the nose (Spanel et al. 2013). Estimated FA deposition in the upper airways and DPX formation were similar in children and adults (c.f. WHO 2010; Nielsen and Wolkoff 2010; Nielsen et al. 2013).

Uptake of FA in the nose of rats, monkeys and humans was estimated by means of an anatomically accurate computational fluid dynamics model. At ≥0.1 ppm, the nasal uptake was about 99, 87 and 85 %, respectively. The uptake was nonlinear, especially at lower concentrations (<0.1 ppm), and thus resulted in a lower nasal uptake fraction due to the effects of endogenous FA. Also, the higher fluxes were predicted to occur in regions located in the more anterior sections of the nose (Schroeter et al. 2014).

A mechanistic model was developed to study the uptake of airborne FA and transport into the surrounding lung tissue at 1 mg/m^3^ in humans. Disregarding the scrubbing effects of the nasal and oral tissue, it was predicted that FA would be quickly absorbed (~97 %) by the mucus membranes with a very high uptake in the trachea (airway generation 0), and that no FA would pass beyond airway generation 8. Thus, no FA would reach the deep airways, including the alveoli, and no FA was predicted to pass to the blood compartment (Asgharian et al. 2012).

In the mucus layer, CH_2_(OH)_2_ diffuses into the epithelial cells and liberates FA, which reacts with glutathione (GSH), proteins, DNA and RNA. The GSH adduct (GS-FA) is oxidized by the FA dehydrogenase to the formate adduct. After hydrolysis, GSH and formate are released. Rats were exposed to 0 (control), 0.7, 2, 6, 10 and 15 ppm FA 6 h/day for 1, 4 or 13 weeks. Nasal tissue concentrations of CH_2_(OH)_2_, GSH, GS-FA and DPX were assessed as were histological effects, epithelial cell proliferation and gene expression. The data were analysed by means of a pharmacokinetic model, taking into account the background CH_2_(OH)_2_ and GSH levels. The cellular levels of CH_2_(OH)_2_ and DPX only showed a minor increase with exposures at 0.7 and 2 ppm FA. At these levels, GSH decreased slightly. Several ppm FA would be required to achieve significant changes. Above 4 ppm, the changes were more conspicuous. Histopathology showed nasal lesions at 2 ppm and epithelial cell proliferation at higher concentrations. The lowest benchmark dose for change of gene expression approximated 1 ppm. The authors concluded that genomic changes at 0.7–2 ppm likely reflected changes in extracellular CH_2_(OH)_2_ and GSH levels and that FA levels below 1 or 2 ppm would not affect FA homeostasis within the epithelial cells (Andersen et al. 2010).

A major advance was the differentiation between FA-induced DNA damage from the (normal) endogenous FA (CH_2_O) level in blood and tissue and from the inhaled (exogenous) FA, using isotope-labelled FA (^13^CD_2_O) for the airborne exposure; exposure in rats was to 10 ppm labelled FA for 1 or 5 days at 6 h/day. Inhaled FA induced labelled mono-adducts (N^2^–HO–^13^CD_2_-deoxyguanosine; labelled FA-dG), DNA–^13^CD_2_–DNA cross-links (labelled dG–FA–dG) and labelled DPX in the nasal tissue. Both at 1 or 5 days of exposure, the labelled FA-dG adduct was about 10 times more common than labelled dG–CH_2_–dG in the nasal tissue. The labelled FA-dG adduct on day 1 and day 5 was 32 and 46 %, respectively, and labelled dG–FA–dG was 45 and 59 %, respectively, of the respective adduct type. Neither labelled FA–dG nor labelled dG–FA–dG was detected in the liver, lungs, thymus, bone marrow, spleen and the blood lymphocytes. In contrast, high amounts of endogenous FA adducts were detected in all tissues. This indicated that exogenous FA exposures only had access to the portal-of-entry area (Lu et al. 2010). The dominating FA adduct to DNA is the FA–dG, which can be used as a sensitive biomarker of FA exposure (Lu et al. 2012a).

A single 6-h exposure to 0.7, 2, 6, 9 or 15 ppm in rats showed that the ratio between exogenous FA–dG and endogenous FA–dG was 0.01, 0.03, 0.2, 0.6 and 2.8, respectively, indicating a strongly nonlinear relationship in the nasal tissue. No exogenous FA–dG adduct was found in the bone marrow at the 15-ppm exposure concentration (Lu et al. 2011). Also in monkeys exposed to 2 or 6 ppm, 6 h/day for 2 days, the external FA–dG adduct was only detected in the nose and not in the bone marrow. At 6 ppm, the FA–dG adduct level was lower in the monkeys than in rats with a single 6-h exposure, suggesting a lower genotoxic effect in primates than in rats (Moeller et al. 2011).

FA is a major source of N^6^-formyllysine (FA-Lys) adducts in cell proteins. In rats, exposure to isotope-labelled FA (^13^CD_2_O) at 0.7, 2, 6 and 9 ppm for 6 h was used to differentiate between adducts from exogenous and endogenous FA-Lys adducts in the total amount, the cytoplasmic, the membrane and the nuclear proteins. After proteolysis and analysis of FA-Lys, the ratio between exogenous and endogenous adducts was shown to increase with increasing exposures; for example, for the total amount nasal epithelial proteins, the ratio was 0.035, 0.14, 0.15 and 0.40, respectively. At each FA exposure, the ratios were in the order cytoplasmic ≈ membrane > soluble nuclear > chromatin protein bound, indicating a decrease in the exogenous FA concentration from the cytoplasmic to the nuclear proteins. In contrast, the endogenous FA-Lys adducts were similar at all exposure concentrations in all cellular compartments. Moreover, this indicated that the exogenous FA exposure did not influence the endogenous FA production. No external FA-Lys adducts were detected in the lungs, liver and bone marrow, and thus the results paralleled studies on FA-dG adducts, confirming that direct exogenous FA effects are limited to the nasal epithelium (Edrissi et al. 2013).

In rats, absorption of inhaled FA into the blood was studied with (^13^C) labelled FA for a single 6-h exposure to 10 ppm; this allows differentiation between endogenous FA and FA from external exposure. The background blood FA levels were from 1.9 to 5.4 mg/L. Inhalation of FA did not increase the blood FA level nor was inhaled (^13^C labelled) FA detected in the blood above the natural background level (Kleinnijenhuis et al. 2013). These findings provide further support for the finding that the airway epithelium in rats is an efficient barrier against even high FA concentration and its transport into the blood.

In a recent rat study, the exposure period was extended to 28 days with 2 ppm (^13^CD_2_)-labelled FA for 6 h per day and 7 days per week. Exogenous and endogenous FA–DNA adducts were obtained from the labelled and unlabelled FA–dG biomarker. The biomarker was considered to represent both mono-adducts and DPX-adducts as the DPX cross-links hydrolysed spontaneously to the mono-adduct. The exogenous adduct accumulated during the 28-day period and reached quasi-steady state after 28 days, at which point the ratio between the exogenous and endogenous adducts was 0.37 in the nasal tissue; this value was higher than the ratio after a few exposures. In the first 6 h post-exposure, there was a rapid initial loss of nearly 20 % of the adducts in the nasal tissue that was followed by a phase with a longer half-life of 7.1 days. This was considered to reflect DNA repair and/or spontaneous hydrolysis. No consistent exogenous adducts were found in internal organs, including the white blood cells, trachea, tracheal bronchial lymph nodes and lungs. This is in agreement with results from previous studies with fewer exposures conducted by the research group. Also, monkeys (cynomolgus macques) were exposed to ^13^CD_2_-FA at 6 ppm for 6 h per day for 2 days. The exogenous biomarker was only observed in the nasal tissue and not in the tracheal carina, proximal trachea, white blood cells and the bone marrow (Yu et al. 2015).

Furthermore, DPX formation has been studied by an ultrasensitive and selective liquid chromatography-mass spectrometry method, where monkeys and rats were exposed to (^13^CD_2_)-labelled FA. This allowed differentiation between DPX from inhaled FA and DPX from endogenous (normal) FA. Monkeys were exposed at 6 ppm, 6 h per day for 2 days. Labelled DPX was detected in the nasal tissue, but not in the peripheral blood mononuclear cells, bone marrow and the liver. Endogenously generated DPX was detected in all investigated tissues. In the nasal tissue, endogenous DPX was about threefold higher than the exogenously generated DPX. Different tissues had different endogenous DPX levels. Thus, endogenous DPX was almost threefold higher in the liver than in the nasal tissue. Rats were exposed at 15 ppm, 6 h per day up to 4 days. Also in rats, exogenous DPX was only detected in the nasal tissue. Furthermore, the decay of exogenous DPX was studied in rats, which were exposed at 2 ppm, 6 h per day for 7 and 28 days, respectively, with a post-exposure period up to 7 days. In the post-exposure period, the exogenous DPX decreased slowly (~10 %). In the nasal tissue, exogenous DPX increased with the number of exposures in both rat studies (Lai et al. 2016). It is noted that inhaled FA only caused DPX formation in the nasal tissue and DPX formation in internal organs cannot be explained by a direct transport of FA to the internal organs.

Overall, the recent studies have demonstrated that airborne FA does not reach internal organs. Thus, if systemic effects occur, they have to be explained by secondary effects from portal-of-entry toxicity, which includes sensory-irritation-induced hypoxia (Nielsen et al. 2013) and airway inflammation. Other important findings are that the recent studies confirm that the external-induced FA-DNA adducts increase disproportionately in the nasal tissue at high FA concentrations that is similar to the exposure–response relationship for nasal cancer in rats. Furthermore, rats had more exogenous induced DNA adducts in the nasal tissue than monkeys.

The WHO (2010) IAQG accepts that direct internal organ effects may occur if the metabolic capacity of the upper airways is overloaded; this may begin at ≥2 ppm. Overall, the WHO (2010) evaluation constitutes a conservative approach.

## Genotoxicity

Formaldehyde is genotoxic due to its covalent binding to DNA, causing DNA mono-adducts, DNA–DNA cross-links, DPX and DNA glutathione cross-links that can cause mutations and clastogenic effects such as DNA strand breaks, chromosomal aberration (CA), micronucleus (MN) formation and sister chromatid exchange (SCE) as reviewed (IARC 2006; RAC 2012; NRC 2014; Kawanishi et al. 2014; Yu et al. 2015). Repair of the FA–DNA mono-adducts may include the base excision repair (BER) pathway, and the intra-strand cross-links may be by the nucleotide excision repair (NER) pathway (Kawanishi et al. 2014). FA-induced DPX may be repaired by the NER repair and by the homologous recombination (HR) pathways (de Graaf et al. 2009; Kawanishi et al. 2014; McHale et al. 2014). Furthermore, DPX may partly be broken down by specific proteolytic enzymes, allowing translesion synthesis polymerases (a potentially mutagenic pathway) to replicate across DNA-peptide lesions. Additionally, a tolerance pathway also exists, allowing replication across unrepaired DPX lesions that may include strand breaks (potentially causing genomic rearrangements) followed by strand ligation (Stingele et al. 2015). Not least, the Fanconi anaemia pathway is important in the repair of inter-strand DNA cross-links and DPX (Ren et al. 2013; Kirsch-Volders et al. 2014; McHale et al. 2014; Schneider et al. 2015).

### Endogenously generated FA and toxicity

As FA is an endogenously generated compound, it may play a role in induction of diseases. Thus, a recent experimental study showed that elevation of the endogenous (natural) FA concentration in tissues can cause cell damage and destruction, as well as genetic damage and cancer (Pontel et al. 2015). As FA is detoxified to formate by the alcohol dehydrogenase 5 (ADH5), mice without the gene (*Aldh5* ^−*/*−^) had elevated FA-dG adducts in the bone marrow (1.7-fold), kidney (1.7-fold) and liver (2.3-fold) compared with the wild-type (*Ald5* ^+*/*+^) mice. In *Aldh5* ^−*/*−^ mice, administration of methanol [a FA precursor (Lu et al. 2012b)] further increased the level of FA–dG adducts. As the FANCD2 protein is involved in the repair of FA–DNA cross-links, *Fancd2* ^−*/*−^ mice were also studied. The double deletion (*Aldh5* ^−*/*−^ *Fancd2* ^−*/*−^) caused a profound decrease in survival, induced blood pancytopenia, reduced bone marrow cellularity (including hematopoietic stem and progenitor cells) and colony formation at cultivation of spleen hematopoietic stem cells. Cultivated spleen B cells stimulated with lipopolysaccharide showed a high level of chromosome breakages. Additionally, liver and kidney dysfunction with DNA damages were also observed. In contrast, no or limited effects were observed on the mentioned endpoints in the wild-type mice or mice with a single deletion of *Aldh5* ^−*/*−^ or *Fancd2* ^−*/*−^, indicating a profound synergistic interaction between deletion of both the *Aldh5* ^−*/*−^ and *Fancd2* ^−*/*−^ genes. Transplantation of bone marrow from the wild-type mice to the double-deficient (*Aldh5* ^−*/*−^ *Fancd2* ^−*/*−^) mice increased survival time and decreased kidney toxicity, but these animals developed hepatocellular- and cholangiocarcinoma as well as T-lymphoblastic leukaemia. The authors concluded that FA is an important source of endogenous DNA damage that is counteracted in mammals by conserved protection mechanisms. It is noted that FA can cause serious damage at the place of contact. However, to observe these effects, external FA has to reach the blood and afterwards the internal organs, and this has not been observed in comprehensive toxicokinetic studies.

### Genotoxicity in human epithelial and blood cells

Previous reviews have shown that occupational exposures, either to mean or to peak FA concentrations from about 1 ppm and above, were associated with single strand break, MN formation, SCE and chromosomal aberration in buccal and nasal epithelial cells, and in peripheral lymphocytes (Nielsen and Wolkoff 2010; Nielsen et al. 2013). This indicates that an IAQG has to be below 1 ppm.

Ladeira et al. (2013) studied genotoxicity in buccal mucosa cells and showed an increase in MN frequency in employees exposed to FA in six histopathology hospital laboratories in Portugal; mean exposure for 8-h periods was 0.16 ppm (range 0.04–0.51 ppm) with a mean peak exposure of 1.14 ppm (range 0.18–2.93 ppm). The buccal MN effect may be a high-level effect. This finding was supported by a previous study with exposure for 4 h per day for 10 working days, where the daily background (constant) FA exposures ranged from 0.15 to 0.5 ppm with added peak exposures up to 1 ppm. At these exposure levels, no increase was observed in buccal MN compared to the pre-exposure MN level (Speit et al. 2007). Similarly in a controlled chamber study with FA exposure for 4 h for 5 days, where the FA concentrations ranged from 0.3 to 0.7 ppm with peaks up to 0.8 ppm, FA exposure had no effect on MN occurrence in the nasal epithelium (Zeller et al. 2011).

The recent studies on genotoxic effects in blood lymphocytes are listed in Table 1. Studies in pathology laboratories confirm the previous association between FA exposure and genotoxicity in lymphocytes, where exposures included mean FA concentrations or peak concentrations above 1 ppm. Where peak exposures were not reported, it can reasonably be assumed that exposures included peak concentrations above 1 ppm, as suggested from the studies where peak exposures were measured and in agreement with previous evaluations (Nielsen and Wolkoff 2010; Nielsen et al. 2013). Furthermore, this is supported by a study in pathology laboratory workers, who were exposed during successive decanting operations, where they manually emptied and filled tissue processor reagent reservoirs (Persoons et al. 2012). The measured 15-min average concentration was 1.17 mg/m^3^ (1.0 ppm), whereas the estimated concentration was 1.7 mg/m^3^ (1.4 ppm), and the upper 95th percentile was 4.32 mg/m^3^ (3.5 ppm). The mean instantaneous peak concentration was 19.5 mg/m^3^ (16 ppm), and the upper 95th percentile was 43.4 mg/m^3^ (35 ppm). In another study, plywood workers had exposures to high mean concentrations (Lin et al. 2013), and the peak concentrations may reasonably have been considerably higher than the mean concentrations. In contrast, workers in a medium density fibreboard plant (Aydin et al. 2013) had a stable exposure concentration as the mean and the peak concentrations were of the same order of magnitude (≤0.3 ppm). However, the interpretation of the study by Aydin et al. (2013) is complicated by the fact that 57 % of the workers used dust masks, suggesting a considerable and unmeasured dust concentration. It is also noted that positive comet assay tests with increased DNA migration were observed in some studies (Lin et al. 2013; Costa et al. 2015), which is difficult to reconcile with a direct FA effect as FA should induce DPX, causing a decrease in DNA migration (e.g. Speit et al. 2009).

**Table 1:** Recent studies on cytogenetic effects in peripheral blood lymphocytes in formaldehyde (FA)-exposed employees

Exposure	Number of participants; exposed (*E*), non-exposed controls (*C*), internal control group [*C* (int)] and smokers (*S* %)	FA exposure in years, mean (range) or as indicated	Exposure in ppm: mean (*M*) (range) peak (*P*) (range) or as indicated	Statistically significant association with FA exposure
Pathology laboratories (Costa et al. 2015)	*E*: 84 (*S*: 25) *C*: 87 (*S*: 25)	12 (SD 8.2)	*M*: 0.38 (0.08–1.39) (8 h TWA) *P*: (0.3–3.2)	CA: increased Aneuploidy: increased Comet: pos
Anatomy and Forensic medicine laboratories (Souza and Devi 2014)	*E*: 30 (*S*: 50) *C*: 30 (*S*: 33)	10.7 (1–30)	*M*: ? *P*: ?	MN: increased
Pathology laboratory (Bouraoui et al. 2013)	*E*: 31 (*S*: 10) *C*: 31 (*S*: 13)	15.7 (SD 10.1)	*M*: ? (0.2–3.4) *P*: ?	MN: increased Aneuploidy: increased
Pathology laboratories (Costa et al. 2013)	*E*: 35 (*S*: 20) *C*: 35 (*S*: 20)	12.5 (1–30)	*M*: 0.36 (0.23–0.69) *P*: ?	MN: increased SCE: increased
Histopathology laboratories (Ladeira et al. 2013)	*E*: 54 (*S*: 20) *C*: 82 (*S*: 31)	?	*M*: 0.16 (0.04–0.51) (8 h TWA) *P*: 1.14 (0.18–2.93)	MN: increased NPB: increased NBUD: increased
Pathological departments (Musak et al. 2013)	*E*: 105 (*S*: 28) *C*: 250 (*S*: 19)	14.7 (SD 10.4)	*M*: 0.32 (0.14–0.66) *P*: ?	CA: increased
Plywood workers (Lin et al. 2013)	E (high): 38 (*S*: 32)	2.52 (SD 2.0) for all in the high, low and int. groups	*M*: 1.20 (0.74–1.66) (8 h TWA) *P*: ?	Comet: pos DPX: NS MN: NS
	E (low): 58 (*S*: 29)		*M*: 0.55 (0.37–0.64) (8 h TWA) *P*: ?	Comet: pos DPX: NS MN: NS
	C (int): 82 (*S*:40)		*M*: 0.11 (0.015–0.20) (8 h TWA) *P*: ?	
	*E*: 62 (*S*: 18)	Effect across an 8 h workday	*M*: 0.22 (0.01–0.54) (8 h TWA) *P*: ?	Comet: pos DPX: increased, but not FA dependent MN: NS
Medium density fiberboard plants (Aydin et al. 2013)	*E*: 46 (*S*: 39) *C*: 46 (*S*: 50)	7.3 (0.33–30)	*M*: 0.2 (0.10–0.33) *P*: ≤ 0.35	Comet: significantly lower than in the controls

*CA* chromosomal aberration, *Comet* comet assay and *pos* positive for genotoxicity, *DPX* DNA–protein cross-links, *MN* micronucleus, *NBUD* nuclear buds, *NPB* nucleoplasmic bridges, *NS* not significant, *SCE* sister chromatid exchange, *TWA* time-weighted average exposure, *?* unknown concentration

A cross-sectional study was performed in 43 FA-exposed workers and 51 matched controls (Zhang et al. 2010). The 8-h time-weighted average (TWA) FA concentration was 1.28 (10th, 90th percentile: 0.63, 2.51) and 0.026 (0.009, 0.026) ppm, respectively. The FA-exposed workers had a significantly lower white and red blood cell, lymphocyte, granulocyte and platelet count, but not of monocyte count. Blood mononuclear cells were cultivated to granulocyte–macrophage colony-forming progenitor (CFU-GM) cells, which were 20 % lower in the FA-exposed workers. However, this was not statistically significant (*p* = 0.10). A small subset of 10 workers with a high TWA-FA concentration [2.14 (1.38–4.14) ppm] was compared with 12 controls [0.026 (0.015–0.026) ppm]. In the CFU-GM cells, monosomy for chromosome 7 increased from about 5 % in the controls to about 10 % in the FA-exposed, and trisomy for chromosome 8 increased from about 4 % to about 12 %, respectively. As all participants had cultivation to CFU-GM cells, this allowed investigation of aneuploidy and structural chromosome aberrations in an expanded subset of 29 workers and 23 controls with a TWA-FA concentration of 1.38 (0.78, 2.61) ppm and 0.026 (0.015, 0.026) ppm, respectively. Monosomy was significantly increased for 16 of 24 chromosomes with the highest significance for chromosomes 1, 5, 7, 4 and 19, shown in decreasing order of significance. Trisomy was significantly increased for 6 of 24 chromosomes, which were chromosomes 5, 19, 21, 1, 20 and 16. Tetrasomy was significantly increased for 10 of 24 chromosomes, with the highest significance for chromosomes 4, 15, 17, 14 and 3. Structural chromosome aberration was only detected for chromosome 5 (Lan et al. 2015). It is noted that the two studies are subsets from the same cross-sectional study with high FA exposures. It is a reasonable assumption that the peak exposures may have been much higher. Further, it is unlikely that FA reaches the internal organs, including the bone marrow and the bone marrow progenitor cells. Also, the studies are not consistent with in vitro effects of FA as reviewed below.

Blood from healthy young non-smoking volunteers were used to derive CFU-GM cells, which were investigated for monosomy and trisomy from chromosomes 5, 7 and 8. The frequency of aneuploidy metaphases was similar and low for the three chromosomes and not increased by exposure to 10–50 µM FA during the cultivation. In contrast, vincristine (an aneugen) increased monosomies for all three chromosomes, but caused no clear increase in trisomy (Kuehner et al. 2012). This was further supported by an in vitro study in FA-exposed TK6 cells, where gene expression was analysed using a whole-genome microarray. This showed that the gene expression profile in FA-exposed cells was closer to the two clastogens, methyl methanesulfonate and ethyl methanesulfonate, than to the two aneugens, colcemid and vincristine (Kuehner et al. 2013). Additionally, human peripheral blood mononuclear cells were cultivated to derive erythropoietic progenitor cells, which were exposed to 0 (control group), 50 or 100 µM FA in vitro. The FA exposure did not significantly increase monosomy or trisomy for chromosome 7 and 8, respectively, even though a high number of cells (~7000) were investigated. Nevertheless, combining monosomies for chromosomes 7 and 8 increased the frequency from 1.45 % in the control group to 1.93 % (statistically significant) in the 50 µM group, but the frequency was decreased to 1.24 % in the 100 µM group. Combining trisomies for chromosomes 7 and 8 resulted in a frequency of 0.03 %, 0.13 % (statistically significant) and 0.09 % (not significant), respectively (Ji et al. 2014). It is noted that the frequencies of aneuploidies were much lower than in the Zhang et al. (2010) and Lan et al. (2015) studies.

Overall, several recent studies confirm a genotoxic effect of FA in blood lymphocytes; the genotoxic effects indicate risk of developing malignant diseases (e.g. Norppa et al. 2006; Kirsch-Volders et al. 2014). However, with regard to assessing risk, previously conducted studies are still the most informative for setting an IAQG for FA. Thus, in a controlled chamber study with volunteers exposed to FA for 4 h per day for 5 days, where the FA concentrations ranged from 0.3 to 0.7 ppm with peaks up to 0.8 ppm, no relevant genotoxic effect was found in blood lymphocytes (Zeller et al. 2011). In contrast to the results from human studies, rats exposed for 6 h per day, 5 days per week for 4 weeks at FA concentrations up to 15 ppm showed no genotoxic effects in peripheral blood cells in the Comet, MN and SCE tests (Speit et al. 2009). Overall, an IAQG has to be below 1 ppm for both mean and peak FA exposures as previously suggested (Nielsen and Wolkoff 2010; Nielsen et al. 2013). Genotoxic effects can be used as a proxy for the risk of malignant diseases, but because comprehensive long-term epidemiological and animal studies are available with the ultimate endpoints, these studies together with the toxicokinetic studies should constitute the appropriate and final basis for evaluation of the WHO guideline value on cancer risk assessment.

### Genetic polymorphisms and genotoxicity in human blood cells

The study by Pontel et al. (2015) identified potential pathways in which polymorphisms in humans may give rise to especially sensitive individuals that must be considered in the guideline setting. ADH5 (also termed ALD3) is the key FA-metabolizing enzyme (Staab et al. 2008). Expression of mRNA of ADH5 in peripheral leucocytes was not affected by FA exposures in a controlled chamber study with 4 h of FA exposure per day for 5 days with FA concentrations between 0.3 and 0.7 ppm and with peaks up to 0.8 ppm (Zeller et al. 2011). However, 38 single nucleotide polymorphisms (SNPs) were described that are nearly all rare (<1 %) alleles, in agreement with two of the polymorphisms (rs11568816, investigated in 150 subjects, and 17028487, investigated in 70 subjects) that showed no variant allele. As regards the third polymorphism (rs13832), 41 % of alleles were heterozygous (G/T) and 59 % were homozygous (44 % T/T and 15 % GG). These polymorphisms had no influence on the expression of blood cell mRNA of ADH5, and FA exposure of cultivated blood cells showed no difference in DPX levels (Just et al. 2011). In a recent study, pathology laboratory employees with a mean peak FA exposure of 1.14 ppm and with a maximum of 2.91 ppm were investigated for genotoxic effects of FA in the cytokinesis-block micronucleus assay. Two ADH5 polymorphisms (Val309Ile and Asp353Glu) did not show biologically relevant effects on FA-induced genotoxicity (Ladeira et al. 2013).

Numerous studies have shown that polymorphisms involved in DNA repair and metabolism influence genetic damage in human peripheral blood lymphocytes (e.g. Dhillon et al. 2011). Costa et al. (2015) investigated polymorphisms of three genes, *XRCC1, PARP1* and *MUTYG*, which participate in the BER pathway (Dhillon et al. 2011), and *XRCC3*, which participates in the HR pathway (de Oliveira et al. 2014); mean exposures were up to 1.39 ppm and peak exposures up to 3.2 ppm. The investigated endpoints included CAs, aneuploidies, aberrant cells, multi-aberrant cells and percentage of DNA in the comet tail. The *XRCC1* allele rs1799782 (Arg194Trp) was associated with more DNA in the tail (damage) in the heterozygous (Arg/Trp) than in the homozygous (Arg/Arg) wild type; none of the other endpoints showed an association with this allele. The authors mention that the effect was only observed in the heterozygous group and the group contained a small number of FA-exposed individuals. The *PART1* allele rs1136410 had lower occurrence (protective effect) of multi-aberrant cells in the heterozygous ((Val/Ala) type than in the homozygous (Val/Val) wild type. None of the other investigated alleles (*XRCC1* rs25487, *MUTYH* rs3219489 and *XRCC3* rs861539) showed any significant association with the FA-induced effects in the investigated endpoints. It is noted that a high number of statistical tests were conducted and that this may have caused mass significance. In previous studies, the *XRCC3* allele with the same polymorphisms was investigated in the cytokinesis-block micronucleus assay with MN, nucleoplasmic bridges and nuclear buds (NBUD) as the endpoints (Ladeira et al. 2013), where the Thr241Met had a higher frequency of NBUD formation. It is noted that no increase was seen in the two other endpoints or in any of the endpoints studied in the recent investigation by Costa et al. (2015). In a study by Costa et al. (2008), polymorphisms in *ERCC1* allele rs3212986*, ERCC4* rs180067, *ERCC5* rs17655 and *ERCC5* rs2227869 were investigated, which are all genes involved in the NER pathway (Dhillon et al. 2011); mean exposures were up to 1.58 ppm, and peak exposures up to 4.43 ppm. The investigated endpoints were MM, SCE and the comet tail length. The authors did not find any effect in these endpoints.

Several phase I and phase II metabolizing enzymes have also been investigated for effects of polymorphisms on FA-induced genotoxicity. Cytochromes P450 (CYPs) are phase I mono-oxygenase enzymes, where CYP2E1 is involved in metabolism of many carcinogenic and non-carcinogenic compounds (Trafalis et al. 2010). The genotoxicity of FA was investigated in blood lymphocytes of FA-exposed subjects with a *CYP2E1* polymorphism (rs6413432) with the wild type carrying the T/T allele versus the combined T/A plus A/A allele group. CAs were not affected by the alleles, whereas the T/A plus A/A allele group had a lower amount of DNA in the comet tails; the authors suggested that this represented a protective effect (Costa et al. 2015).

Glutathione *S*-transferases (GSTs) are phase II enzymes that catalyse conjugations of glutathione to electrophilic centres of reactive compounds. Polymorphisms of the *GSTM1*, *GSTT1* and *GSTP1* genes have been associated with lymphohaematopoietic malignancies or predisposition to these (Dahabreh et al. 2010; Bin and Luo 2013; He et al. 2014). Comparing FA-induced genotoxicity in the *GSTM1* null versus in the *GSTM1* non-null and in the *GSTT1* null versus in the *GSTT1* non-null polymorphisms, respectively, showed no consistent difference between the respective null and non-null genotypes (Costa et al. 2008; Jiang et al. 2010; Santovito et al. 2011; Zeller et al. 2012; Costa et al. 2015). Furthermore, FA-associated genotoxicity was investigated in the *GSTP1* gene, where the isoleucine (Ile) amino acid at position 105 in the wild type (Ile/Ile) was substituted with valine (Val) with the heterozygous (Ile/Val) genotype and the mutant (Val/Val) genotype. Whereas FA-associated CAs were lower in the combined Ile/Val plus Val/Val group than in the Ile/Ile group (Costa et al. 2015), no effect was observed in the comet assay (Jiang et al. 2010; Costa et al. 2015), and MN was marginally (~26 %) increased (Jiang et al. 2010).

In conclusion, no major influence of polymorphisms has been identified that constitutes an important risk factor with regard to the genotoxic effects of FA exposure.

### Oxidative stress-associated genotoxicity in animal studies

Reactive oxygen species (ROS) may be caused by air pollutants (Azad et al. 2008), for example as a consequence of exposure to inhaled particles and their associated compounds (Møller et al. 2014). Reactive oxygen species (ROS), such as superoxide anion, hydrogen peroxide, hydroxyl radical and hydrochlorous acid, and reactive nitrogen species (RNS), such as nitric oxide and peroxynitrite, are highly reactive towards lipids, proteins and DNA (Azad et al. 2008; Filomeni et al. 2015) that may cause cellular adaptation by up-regulation of antioxidant and repair mechanisms or cell death and cancer (Azad et al. 2008; Filomeni et al. 2015).

In vitro exposure of the lung A549 epithelial cell line to FA caused DPX formation, malondialdehyde formation and up-regulation of DNA transcription factors (NF-ĸB and AP-1). Moreover, it also caused a decrease in superoxide dismutase and glutathione peroxidase, which were attenuated by antioxidants such as curcumin (Zhang et al. 2013a) and selenium (Shi et al. 2014), indicating that genotoxicity and ROS formation can play a direct role in FA-induced cellular toxicity. Recent studies in mice and rats of the association between FA exposures and DNA damage and ROS effects are evaluated below.

Acute 2–24 h of exposure at 0 (control) and 0.1 ppm FA were studied in ICR mice. The marker, 8-hydroxy-2′-deoxyguanosine, of ROS-induced DNA damage was unchanged in urine, plasma, lungs, liver and brain in the FA-exposed animals. NO production was measured after conversion to the stable NO_3_^−^ ion. Plasma NO_3_^−^ levels were only increased after 24 h of exposure. The lung NO_3_^−^ level was unaffected by the exposures. In contrast, the liver and brain NO_3_^−^ levels decreased after ≥2 h of exposure, and the urinary NO_3_^−^ levels decreased after ≥8 h of exposure. Plasma interleukin-6 (IL-6) was unaffected by the exposures, and no tissue damage was observed. However, exposure at 3 ppm for 24 h increased superoxide dismutase in urine and plasma, but not in the liver (Matsuoka et al. 2010). It is noted that the low FA concentration did not show consistent adverse effects, including oxidative stress.

A recent comprehensive study in BALB/c mice evaluated DPX formation, decrease in glutathione (GSH), increase in ROS, and increase in malondialdehyde (MDA) formation in bone marrow, peripheral blood mononuclear cells (PBMC), lungs, liver, spleen and testes. Mice were exposed to 0 (control), 0.5, 1 and 3 mg/m^3^ FA 8 h/day for 7 consecutive days. The liver was the most sensitive organ for DPX formation, decrease in GSH and increase in ROS (LOAEC: 0.5 mg/m^3^). The NOAEC for DPX formation in the bone marrow, spleen and testes was 0.5 mg/m^3^. Least sensitive was the PBMC and the lungs, where no increase in DPX was observed at 3 mg/m^3^. The lung was highly sensitive to a decrease in GSH with a LOAEC of 0.5 mg/m^3^. The bone marrow, PBMC and spleen had an intermediate sensitivity (NOAEC: 0.5 mg/m^3^). The NOAEC for the testes effect was higher (1 mg/m^3^). No ROS formation occurred at 0.5 mg/m^3^ (NOAEC) in the bone marrow, the lungs, spleen and testes. PBMC was less sensitive with a NOAEL at 1 mg/m^3^. For MDA formation, the NOAEC was 0.5 mg/m^3^ for the bone marrow, lungs, liver, spleen and testes effect, whereas the NOAEC for the PBMC was 3 mg/m^3^. To further substantiate the effect of oxidative stress, two groups of mice were exposed to 3 mg/m^3^ FA, where one group was given an additional oral dose of 100 mg/kg GSH after each FA exposure. In the GSH-treated group, bone marrow, PBMC, lungs, liver, spleen and testes had higher GSH levels than in the GSH-untreated group. Furthermore, ROS and MDA formation was lower than in the untreated group. The GSH group had lower DPX formation, except in the lungs, where the level was similar in the GSH-treated and untreated group. It is mentioned that mice reduce their respiratory minute volume, which may induce oxidative stress (Ye et al. 2013). It is noted that the liver was the most sensitive organ (LOAEC: 0.5 mg/m^3^) and the NOAEC was higher (0.5 mg/m^3^) for bone marrow effects.

The finding that FA exposures can cause DPX formation at distant sites was supported by two previous studies of Kunming mice that were exposed continuously for 72 h at the above-mentioned concentrations. DPX formation was increased significantly in the bone marrow (Cheng et al. 2010) and the kidney and testes (Peng et al. 2006) at 0.5 mg/m^3^ (LOAEC), whereas the NOAEC for DPX formation in the liver was 0.5 mg/m^3^ (Peng et al. 2006).

In another recent study, male BALB/c mice were exposed to 0 (control), 0.5 and 3 mg/m^3^ FA, 8 h/day, 5 days/week for 2 weeks (Zhang et al. 2013b). At the end of the study, the red blood cell counts were decreased concentration dependently (17 and 27 %, respectively) as were the white blood cell count (43 and 52 %, respectively). The decrease in lymphocytes was similar (~40 %) in the exposed groups. The increase in platelets was 109 and 67 %, respectively. No change was observed in neutrophilic granulocytes and intermediate cells. Bone marrow histology qualitatively suggested that the number of megakaryocytes (producing thrombocytes) increased with increasing FA concentrations. At 3 mg/m^3^, myofibrosis was observed. Bone marrow ROS increased concentration dependently, and the increased ROS was observed already at 0.5 mg/m^3^. At 3 mg/m^3^, a significant decrease was observed in bone marrow GSH and glutathione *S*-transferase theta 1 (GSTT1), whereas a significant increase was observed in cytochrome P450 1A1 (CYP 1A1), NF-ĸB, TNF-α, IL-1β and caspase-3 activity. It is noted that the LOAEC was 0.5 mg/m^3^ with haematological changes, increased bone marrow megacaryocytes and increased ROS formation.

ICR mice were exposed at 0 (controls), 20, 40 and 80 mg/m^3^ (0, 16, 33 and 65 ppm, respectively) for 2 h per day for 15 days. White blood cell and platelet counts were decreased at ≥40 mg/m^3^, whereas the red blood cell count was unaffected at 80 mg/m^3^. In the bone marrow, the most sensitive endpoints were a decrease in superoxide dismutase and an increase in MDA (both oxidative stress markers), which were significant at 20 mg/m^3^ (LOAEC). At ≥40 mg/m^3^, the bone marrow content of Bax and cytochrome *c* (both pro-apoptotic) increased, whereas the Bcl-2 protein (anti-apoptotic) decreased. In the bone marrow at 80 mg/m^3^, a decrease occurred in nucleated cells, in mitochondrial membrane potential and in colony formation at in vitro cultivation. Also at 80 mg/m^3^, there was arrest in the *S* phase of the cell cycle (Yu et al. 2014). It is noted that the FA concentrations are at extremes. They are high compared with the mucosal detoxification mechanisms, and an extreme decrease [>60 %, calculated from Nielsen et al. (1999)] in respiratory ventilation is predicted. This study required high FA concentrations (≥40 mg/m^3^) for change of the blood constituents, which is different from the above-mentioned studies.

Rats were exposed 8 h per day, 5 days per week for 4 or 13 weeks at 10 and 20 ppm FA, respectively. In the heart, the superoxide dismutase activity increased significantly in all exposed groups. The catalase activity decreased significantly in the 4-week groups, but the decrease was not significant in the 13-week groups. The heart lipid peroxidation (thiobarbituric acid reactive) products increased non-significantly in the FA groups. The heart NO levels were not affected by the FA exposures (Güleç et al. 2006). This study applied very high FA concentrations and, at most, it showed a marginal and non-significant increase in ROS formation.

Wistar rats were exposed to 0 (control), 0.5, 1 and 3 mg/m^3^ FA continuously for 72 h. In the bone marrow, DPX was decreased by 14 % at 0.5 mg/m^3^, unaltered at 1 mg/m^3^ and significantly increased by 26 % at 3 mg/m^3^ when compared with the control group. In the comet assay, the percentage of DNA in the tail increased significantly to 250 % at 0.5 mg/m^3^ and to 455 % at 1 mg/m^3^, but no change occurred in the 3 mg/m^3^ group. The tail moments showed a similar pattern (Wang et al. 2009). The slight increase in the DPX formation in the 3 mg/m^3^ might support a slight oxidative stress response associated with a decrease in respiratory ventilation, although rats are less prone to decrease ventilation compared with mice (Nielsen et al. 2013). As FA causes DPX formation, DNA migration in the comet assay is expected to decrease and not to increase (Speit et al. 2009). It is noted that the bell-shaped response in the comet assay is unexplained, as it is not related to an increased oxidative stress or to a potential systemic absorption of FA as no effect was observed at 3 mg/m^3^. It is counter-intuitive that the bell-shaped relationships are relevant proxies for carcinogenic effects, as these effects increase monotonously with exposure concentrations in animal and human studies as discussed below.

In a previous study, rats were exposed 6 h per day, 5 days per week for 4 weeks at FA concentrations up to 15 ppm and showed no genotoxic effects in peripheral blood cells in the Comet, MN and SCE tests (Speit et al. 2009).

For risk assessment, it is noted that the observed effects occurred after short-term exposures and effects show high variability within mice studies as well as between mice and rat studies. The Ye et al. (2013) study observed no increase of DXP formation in PBMC at 3 mg/m^3^. This indicates that FA neither reaches the blood compartment in mice nor can be distributed to the internal organs, in agreement with the isotope-labelled FA studies in rats and monkeys (Lai et al. 2016). Overall, this strongly suggests that the DPX formation and the oxidative stress in distant organs are secondary effects of portal-of-entry effects.

Sensory-irritation-induced effects are candidates for a FA-induced portal-of-entry effect. Thus, BALB/c mice experienced a decrease in their respiratory ventilation due to sensory irritation in the upper airways at ≥0.3 ppm (0.37 mg/m^3^). The examples below indicate that hypoxia may cause profound physiological changes; in humans, hypoxia is known *inter alia* from obstructive sleep apnoea. Chronic intermittent hypoxia may cause cardiovascular deterioration in animals and humans that may be due to oxidative stress, systemic inflammation, sympathetic activation, decrease of bone marrow-derived endothelial progenitor cell mobilization, which decreases repair of endothelial injuries, systemic and pulmonary arterial hypertension, and heart failure (Dumitrascu et al. 2013; Wang et al. 2013b; Yin et al. 2012, 2014). In the lungs, hypoxia can induce oxidative stress and inflammation that can cause bronchial vasoconstriction, pulmonary oedema, vascular remodelling and pulmonary hypertension (Araneda and Tuesta 2012). In addition, oxidative stress in the lungs may induce autophagy, which is a catabolic process that regulates turnover of proteins and eliminates damaged organelles and protein aggregates (Malaviya et al. 2014). Furthermore, hypoxia may cause epigenetic effects due to dysregulation of histone methylation (Chervona and Costa 2012).

Estimated from Nielsen et al. (1999), BALB/c mice decrease their respiratory ventilation between 4 and 15 % at 0.4 ppm (0.5 mg/m^3^), suggesting that hypoxia-induced oxidative stress may be a potential secondary effect, also noted by Ye et al. (2013). This agrees with the higher FA concentration needed for DPX formation in rats (Wang et al. 2009). The concentration that depresses the respiratory rate by 50 % (RD_50_) in mice is about 4–8 ppm (Chang et al. 1981; Nielsen et al. 1999), but about 30 ppm in rats (Chang et al. 1981); the corresponding decrease in respiratory ventilation was 47 and 45 %, respectively (Chang et al. 1981). The difference in the FA effect on the respiratory ventilation has toxicological significance. For example, the nasal tissue in mice received half the dose per unit area and time at 15 ppm compared with the dose in rats, which explains the lower frequency of nasal cancer in mice (Barrow et al. 1983). For that reason, a systemic effect of a potentially absorbed dose should also be lower in mice than in rats. However, the opposite is the case with regard to DPX formation in the bone marrow; here DPX formation is associated with the decrease in ventilation. Moreover, in the bone marrow, hypoxia and ROS formation play an important role in regulation of the hematopoietic stem cells, where oxygen sensors (hypoxia-inducible factors) regulate numerous genes controlling cell proliferation and survival, angiogenesis, metabolism and haematopoiesis (Zhang and Sadek 2014; Morikawa and Takubo 2016). Although sensory-irritation-induced decrease in respiratory rate and ventilation in rodents is one of the most commonly used endpoints for the study of irritation of chemicals, numerous other less well-investigated reflex-induced reactions are also caused by sensory irritation. These reactions include decreased heart rate, increased peripheral vasoconstriction, increase in systolic blood pressure, decreased renal blood flow and clearance, and decreased coronary blood flow (Alarie 1973). Additionally, FA induces other sensory-irritation effects such as decrease in body temperature and decrease in total metabolism, indicated by a decrease in CO_2_ production. These effects were more prominent in mice than in rats (Jaeger and Gearhart 1982). On the whole, the observed FA effects are in a concentration range where sensory irritation is present and where one or more of the secondary sensory-irritation effects may play a role in the observed systemic effects; low oxygen supply may cause oxidative stress in humans (Askew 2002), but this may be less important in rats (Nagatomo et al. 2012), suggesting that species differences may exist.

Risk assessment should be based on relevant long-term studies in mice and rats and not on short-term studies in these species. The 2-year study of FA exposure in mice and rats found only nasal cancer at levels up to 14 ppm (17 mg/m^3^) FA where all organ systems were investigated (Kerns et al. 1983). Thus, speculations about the consequences of the internal organ effects in the short-term studies cannot overrule the findings in the long-term studies. The WHO (2010) guideline prevents sensory irritation; this threshold is considered precautionary and is not contradicted by the short-term studies discussed above.

### Transcriptional regulation by microRNAs

MicroRNAs (miRNAs, miRs) are single-stranded oligonucleotides non-coding RNA sequences of about 22 nucleotides. miRNAs are important regulators of gene expression at the posttranscriptional level. This may result in transcriptional repression, mRNA degradation or up-regulation of gene expression (Vrijens et al. 2015).

Epigenetic changes were studied based on expression profiles of 534 miRNAS in the nasal tissue of nonhuman primates. Thus, cynomolgus macaques were exposed to 0 (controls), 2 and 6 ppm FA for 6 h/day for 2 consecutive days. At 2 ppm, three miRNAs (miR-142-3p, miR-145 and miR-203) were significantly decreased. A decrease in miR-142-3p may be related to the expression of genes that increase cell proliferation. At 6 ppm, FA disrupted expression levels of 13 miRNAs, indicating an exposure-dependent effect. The miR-125b was the highest up-regulated miRNA, and thus it was associated with a decrease in apoptosis-related gene expression. The most decreased miRNAs at 6 ppm FA were miR-145 and miR-142-3p (Rager et al. 2013).

miRNA and mRNA expression profiles were also studied in the nasal respiratory epithelium, in white blood cells (WBC), and bone marrow cells in FA-exposed rats. Exposures were with labelled FA (^13^CD_2_O) at 0 (control) or 2 ppm FA for 7, 28 or 28 days followed by a 7-day recovery period. Exposure lasted 6 h per day. Two ppm was selected as this concentration altered gene expression, caused DNA adduct formation, but only caused minimal inflammatory cell infiltration in the nose. Alteration of expression of 84, 59 and 0 miRNAs was observed among 695 miRNAs in the nasal tissue in the three FA-exposed groups. In the WBC, the altered miRNA expression was 31, 8 and 3, respectively. The miRNA expression was not altered in the bone marrow. The miRNA expression profile showed a strong time-dependent and tissue-specific profile. The expression of miRNAs in the nose was associated with down-regulation of tumour-suppressor activity. It was predicted that miRNA regulated up to 35 % of FA-induced transcriptional responses. The miRNA expression changes did not persist in the nose after 7 days of recovery. Expression of mRNA levels of 27,342 genes was also studied in the nose and WBC. FA exposure caused differential expression of 830 and 42 genes in the nose in the 7-day and 28-day groups, respectively. In the 7-day group, 25 % of the FA-responsive transcripts represent olfactory receptors. In WBC, altered gene expression was seen in 96 in the 7-day group and in 130 in the 28-day group. Both in the nose and the WBC, gene expression was time dependent. Of the FA-responsive transcripts in the nose, only 2 % were also responsive to FA exposure in the WBC with 1 % in the same direction of altered expression. Pathway analyses of miRNAs and mRNA profiles revealed a total of 45 pathways associated with FA-induced transcriptomic changes where enrichment of immune system/inflammation signalling was observed both in the nose and in the WBC. From the sensitive biomarker FA-dG, external FA-dG adducts were detected in the nasal DNA, but no external generated FA-dG adducts were found in the WBC DNA, indicating that external FA did not reach the WBC. This led the authors to hypothesize that FA-induced inflammatory signals originating in the nose drive the effects observed in WBC (Rager et al. 2014). Overall, this study supports that the FA-induced effect on the WBC may be a secondary effect from the upper airways as no FA is absorbed beyond the portal-of-entry mucosa.

In three groups, mixtures of FA, benzene, toluene and xylene, were investigated in mice exposed 2 h/day, 5 days/week for 2 weeks and compared with an unexposed control group. Group 1: 3 + 3.3 + 6 + 6 mg/m^3^, respectively; Group 2: 5 + 5.5 + 10 + 10 mg/m^3^, respectively; and Group 3: 10 + 11 + 20 + 20 mg/m^3^, respectively. The bronchoalveolar lavage fluid content of IL-8 increased significantly in the exposed groups with a similar increase (~25 %). In Group 1, lung tissue GSH decreased non-significantly (15 %), whereas a significant increase was observed in the total nitric oxide synthase (25 %) and in the inducible nitric oxide synthase (66 %). In group 2, the values were 29, 72 and 78 %, respectively, and in Group 3, values were 29, 62 and 36 %, respectively. In the lungs in Group 1, 662 miRNAs were down-regulated and 96 were up-regulated, in Group 2, 592 were down-regulated and 68 up-regulated, and in Group 3, 11 were down-regulated and 18 up-regulated. In Group 1, the most significantly up-regulated miRNAs were miR-1187, miR-125a-3p, miR-466c-5p, miR-5105 and miR-3472, whereas the most significantly down-regulated was miR-125b-5p. These miRNAs were biologically linked to cell death, cell adhesion and metal ion transport (Wang et al. 2014). It is noted that the FA concentrations in themselves decrease the respiratory ventilation substantially, which may cause hypoxia. The exposure-dependent effects were limited or absent; the miRNAs were changed minimally at the highest exposure concentration. Additionally, FA is not expected to reach the lungs if inhaled as a gas. Also, it is not possible to deduce effects of gaseous FA due to mixed exposure.

The WHO (2010) IAQG is for gaseous FA exposure with the NOAEL (1.25 mg/m^3^) for nasal epithelial inflammation in rats is used as a critical effect in the setting of the guideline. Thus, there is no contradiction between the guideline value and the new studies, which have been conducted at concentrations in the effect-range.

## Carcinogenicity

### Previous evaluations

The histopathological NOAEL was 1 ppm for damage of the nasal epithelium in rats and monkeys, repeatedly exposed from 6 to 22 h per day (c.f. Nielsen and Wolkoff 2010). This suggested that the FA concentration may be more important for cytotoxicity and cell proliferation than the total FA dose. FA caused nasal squamous cell carcinoma (SCC) that is the critical cancer type in rats. Fischer 344 and Sprague–Dawley rats were more sensitive in developing SCC than Wistar rats, mice and hamsters. Results from four long-term studies with the sensitive rat strains are combined in Table 2, showing an apparent NOAEC for SCC at 2 ppm (WHO 2010; Nielsen and Wolkoff 2010; Nielsen et al. 2013). In rats, epithelial cell damage-induced cell proliferation was shown experimentally to be a key mechanism for development of SCC; in Wistar rats, no SCC could be induced at ≤1 ppm FA, even with induced cell proliferation (Woutersen et al. 1989). The two NOAECs were used in the WHO (2010) risk characterization. In addition to SCC in the rat nose, FA exposure also induced a lower number of (benign) polypoid adenomas at ≥2 ppm FA (RAC 2012). These tumours were not considered in the WHO (2010) evaluation. This is supported by a recent evaluation that concluded that this type of lesion is unlikely to be a pre-stage to the (malignant) SCC (Gelbke et al. 2014).

**Table 2:** Nasal epithelial squamous cell carcinomas (SCC) in combined groups of male and female rats from four long-term inhalation studies^a^ with formaldehyde (FA) exposures

FA (ppm)	Rats with SCC/group size (% with SCC)
0	0/453 (0)
0.3	0/32 (0)
0.7	0/90 (0)
2	0/364 (0) (apparent NOAEC)
6	3/325 (0.9) (apparent LOAEC)
10	20/90 (22)
14	103/232 (44)
15	120/278 (43)

^a^Kerns et al. (1983), Sellakumar et al. (1985), Monticello et al. (1996) and Kamata et al. (1997). For review of details, see Nielsen and Wolkoff (2010) and Nielsen et al. (2013)

In rats and mice, long-term inhalation of FA has not shown convincing development of lymphohaematopoietic malignancies (WHO 2010; Nielsen and Wolkoff 2010; Golden 2011; Rhomberg et al. 2011; RAC 2012). Nevertheless, if such an effect was masked by a high mortality in rats (IARC 2012) and mice (WHO 2010; IARC 2012) due to development of nasal SCC at high exposure levels, the incidence of lymphohaematopoietic malignancies would be much lower than that of SCC in rats, why SCC was considered the more sensitive endpoint (WHO 2010).

Meta-analyses were used to identify associations between FA exposures and cancer (WHO 2010; Nielsen and Wolkoff 2010). Except for one meta-analysis, which has been repeated and updated (Schwilk et al. 2010), none of the other meta-analyses showed any clear association between the different types of cancer and FA exposure. The meta-analysis by Schwilk et al. (2010) used 13 cohort studies and one nested case control study. The relative risk (RR) was selected from the highest exposure group in each study; the intention was to evaluate potential effects of high FA exposures. Furthermore, when RRs from different exposure metrics were given, the value was selected in the order: peak exposure, average intensity, cumulative exposure, exposure duration, and earlier date of hire. The meta RR (95 % CI) was 1.5 (1.1–2.1) for leukaemia, 2.5 (1.4–4.3) for myeloid leukaemia, and 0.95 (0.6–1.5) for lymphatic leukaemia. Combining all exposed individuals into one group, the mRR was 1.07 (0.86–1.32). This suggested that high exposures may cause leukaemia, especially myeloid leukaemia. However, the analysis has been criticized for methodological shortcomings such as not using all available information and choosing highest exposure cut points that vary across the combined studies, which may cause heterogeneity; the homogeneity tests used in the study are considered insensitive. Predictive intervals are recommended instead of confidence intervals, and the findings of elevated leukaemia and myeloid leukaemia risks are far from significant if using these techniques in the data analyses (Morfeld 2013).

Recently, two comprehensive reviews of epidemiological studies on lymphohaematopoietic malignancies were published. Thus, eight case–control and 24 cohort studies were evaluated by Rhomberg et al. (2011), and 17 case–control and 22 cohort studies were reviewed by Checkoway et al. (2012). None of these reviews found any consistent or strong evidence that FA was causally related to any of the lymphohaematopoietic malignancies, including myeloid leukaemia.

### Recent evaluations by national or international panels

A recent joint EU evaluation of cancer hazards was performed by RAC (2012). After long-term inhalation in rats and mice, nasal SCC and benign tumours (papillomas and adenomas) were the key effects. Moreover, RAC evaluated a series of epidemiological studies, including their strengths and weaknesses, and found the key effect of exposure to be nasopharyngeal cancer (NPC). Based on the overall consistency within and between species, and biological plausibility (comprising all genotoxic effects of FA), RAC concluded that there is “limited evidence of carcinogeneity in humans (Car. 1B)”; the human evidence was from nasopharyngeal cancer. With regard to inhalation, RAC did not find evidence of tumours outside the respiratory tract.

A different conclusion was reached by NRC (2014), which found that there was clear and convincing epidemiological evidence (*Sufficient evidence*) of a causal relationship between FA exposure and occurrence of nasopharyngeal and sinonasal cancer, and myeloid leukaemia; the carcinogenic effect at any additional sites does not meet the requirement of limited evidence. *Sufficient evidence* was accepted if at least two strong or moderately strong studies with different study design and populations showed an association between FA exposure and a specific cancer type and for which chance, bias and confounding could reasonably be ruled out. An epidemiological study was considered strong if it comprised a large population with long duration of exposure and sufficient follow-up for latency, had an appreciable FA gradient, and the FA exposure was well characterized. Accept of a systemic carcinogenic effect does not require that the mechanism is known or FA is systemically available. Also, the negative findings did not necessarily negate positive findings. It is mentioned that the evaluation is hazard based and not a risk assessment. It is noted that limitations of the key studies were not addressed, although they have been discussed intensively in the scientific literature. The different conclusions between the two evaluations are due to differences in evaluation criteria. All the recent studies that were considered to be strong by NRC (Beane Freeman et al. 2009, 2013; Hauptmann et al. 2009; Meyers et al. 2013) are considered below.

### Identification of studies with quantitative exposure response relationships

Quantitative FA exposures and associations with different types of cancer are available from three major and recently updated occupational cohorts: the National Cancer Institute (NCI) cohort (Beane Freeman et al. 2009, 2013), the British (UK) factory cohort with exposures to FA (Coggon et al. 2014), and the US NIOSH Garment Industry cohort (Meyers et al. 2013). Moreover, data were also available from a case–control study of cancer among US embalmers (Hauptmann et al. 2009). The NCI and the UK cohorts are considered to have the best exposure assessments (Checkoway et al. 2012) and thus to be the key studies for establishing exposure–response relationships. The NIOSH cohort had limitations in the exposure assessment (Checkoway et al. 2012), but due to the size of the cohort, it is considered valuable for hazard identification (Table 3). The US embalmer study reported an increase in myeloid leukaemia [OR (95 % CI) 11.2 (1.3–95.6)] where the referent group contained one myeloid leukaemia case who had never been exposed to FA. No increase was observed in the lymphoid malignancies, including Hodgkin’s lymphoma [0.5 (0.1–2.6)]. The study has severe limitations, including lack of exposure–response relationship within FA exposure groups, unstable estimates due to only one case in the referent group, or where more reliable estimates were present, no appropriate statistical testing was provided (WHO 2010; Cole et al. 2010; Golden 2011; Rhomberg et al. 2011). Due to these limitations, the US embalmer study is not considered a key study for risk assessment.

**Table 3:** Cancer risks from formaldehyde exposures in three recently updated occupational cohorts

Study	NCI cohort < 1966–2004			UK cohort (1941–2012)			US garment worker cohort (1955–2008)		
Exposure (ppm)	Median average intensity: 0.3 and range 0.01–4.3. Exposure to ≥ 1 occurred in 15 % and 24 % had peak exposures at ≥ 4			Range <0.1 to >2			Geometric mean: 0.15 and geometric standard deviation 1.90. Past exposures may have been about 4 ppm, see text		
Risk estimate^a^	ICD-8	*O*	SMR	ICD-9	*O*	SMR	ICD-10	*O*	SMR
All cancers	140–209	3146^b^	1.07*	140–208	2241	1.10*	–	1021	0.96
Solid cancers	140–199	2878^b^	**1.09** ^*^	–	–	–	–	–	–
Nose and nasal sinuses	160	3^b^	0.90	–	2	0.71	C30–C31	0	0
Pharynx				–	17	1.20	C09–C14	6	0.88
Nasopharynx	147	9^b^	1.84	–	1 (E: 1.7)	0.59	C11	0	0
Buccal cavity	140–149	74^b^	1.15	–	7	1.08	C03–C08 C46.2	6	1.42
Larynx	161	42^b^	1.23	–	22	1.22	C32	4	0.77
Lung	162	1130^b^	1.14*	–	813	**1.26** ^*^	C33–C34^c^	267	1.04
Bone	170	8^b^	1.36	–	–	–	–		
Prostate	185	261^b^	1.07	–	147	0.80*	–	–	–
Pancreas	157	111^b^	**0.76** ^*^	–	91	1.04	–	–	–
Breast	174	28^b^	**0.64** ^*^	–	–	–	–	–	–
Hodgkin’s disease	201	25	1.42	–	–	–	C81	4	0.95
Non-Hodgkin’s lymphoma	200	94	0.85	–	53	1.06	C46.3	44	1.13
	202						C82–C85		
							C88.0		
							C88.3		
							C91.4		
							C96		
Multiple myelomas	203	48	0.94	–	28	0.99	C88.7	23	1.24
							C88.9		
							C90		
Leukaemia	204–207	116	1.02	–	54	1.02	C91–C95	36	1.04
							Ex91.4		
Myeloid leukaemia	205	44	0.90	–	36	1.20	C92	21	1.28
Lymphatic leukaemia	204	36	1.15	–	–	–	C91–C91.9 Ex 91.4	6	0.71
Stomach	–	–	–	–	182	**1.29** ^*^	–	–	–
Digestive system	150–159	759^b^	1.07	–	126	0.95^d^	–	–	–

The US National Cancer Institute (NCI) cohort comprised 25,619 workers employed in 10 U.S. formaldehyde producing or using facilities. Workers were employed prior to January 1 1966 and were followed up through 31 December 2004 (Beane Freeman et al. 2009; Beane Freeman et al. 2013; number of death was 13,951 [11,346 among exposed and 2605 among unexposed (Beane Freeman et al. 2013)]. Exposures were from Beane Freeman et al. (2009). A British (UK) cohort from six British factories, comprising 14,008 men followed up from 1941 through December 2012; number of death were 7378 (Coggon et al. 2014). The US National Institute for Occupational Safety and Health had established a cohort with 11,034 employees in three garment facilities (US garment worker cohort); number of death was 3915. The study was updated through 31 December 2008 (Meyers et al. 2013)

^a^Standardized mortality ratio (SMR) was obtained by comparison with the national death rates. The number of observed death due to the type cancer among exposed workers is indicated by “O” and the expected number of cases by “E” where relevant for calculation of SMR. When a 95 % confidence interval does not include 1.00, this is indicated by * and bold. Excluded is indicated with Ex. The International Classification of Diseases is indicated by 8th revision (ICD-8), 9th revision (ICD-9) and the 10th (ICD-10). Not given is indicated by “–”

^b^Beane Freeman et al. (2013)

^c^Comprising the trachea, bronchus and lung

^d^Large intestine

In the WHO (2010) evaluation, nasal cancers and lymphohaematopoietic malignancies were the main focus. Additionally, standardized mortality ratios were evaluated for other types of cancer in the three comprehensive occupational cohorts, which have been updated recently (Table 3). A key approach by the WHO (2010) in the evaluations of cancer effects was whether a nonlinear exposure–response relationship could reasonably be identified with a plausible NOAEC. This approach is justified from the nonlinear FA–DNA adduct formation and the assumption that an increase in cancer incidence would occur if the cellular level of FA is increased above the normal background to a level where the detoxification mechanisms are overwhelmed. This causes cytotoxicity and regenerative cell proliferation, during which DNA lesions may be fixed as mutations due to a decrease of time available for DNA repair. Transmission from the normal homeostasis, determining a NOAEC, to a gradual increase in cancer incidence could also be expected to show a nonlinear exposure response relationship. And, importantly, SCC showed a clear, nonlinear exposure–response relationship in rats (Table 2).

### Portal-of-entry cancers in humans

Airway cancers associated with FA exposures were studied in a Finnish cohort with 1.2 million employees. All men born between 1906 and 1945 who were in employment during 1970 were included. The follow-up study was in the Finnish Cancer Register for nasal cancer (292 cases), cancer of the nasopharynx (149 cases) and lung cancer (30,137 cases) during the period 1971–1995. The Finnish job-exposure matrix was used to estimate cumulative exposures. Duration of exposure was estimated from census data. A latency period of 20 years was accepted. Number of exposed cases (*N*), RR obtained by comparison with unexposed, and 95 % confidence intervals were estimated [*N*; RR (95 % CI)]. The RR for FA exposure was adjusted for smoking, socioeconomic status, and exposure to wood dust. The risk of nasal cancer [17; 1.1 (0.6–1.9)], nasal squamous cell carcinoma [9; 1.0 (0.4–2.0)] and nasopharyngeal cancer [5; 0.9 (0.3–2.2)] was not increased. The risk was slightly increased for lung cancer [1831; 1.2 (1.1–1.3)], which was adjusted for asbestos and silica dust exposures. However, the risk in the highest exposure group (FA ≥ 1 ppm) was not increased. Thus, the authors considered the increased risk to be due to residual confounding effects of smoking and co-exposures, including asbestos and crystalline silica. FA exposures were below 1 ppm in most occupations. Only floor layers and men who worked with varnish and lacquer had average exposures at 1 ppm (Siew et al. 2012). Overall, this study found no increase in portal-of-entry cancer at low concentrations of FA in occupational settings, and only a minor part of the nasal cancer cases were associated with FA exposures.

The US National Cancer Institute (NCI) cohort includes 25,619 workers employed prior to 1 January 1966 in 10 industrial plants. The cohort was followed up until 31 December 2004 (Beane Freeman et al. 2013). At present, it is the second largest industrial cohort on FA exposure. In the cohort, 13,951 had died. The median duration of follow-up was 42 years, and median length of employment was 2.6 years. A lag time of 15 years was applied. The calendar year-specific US mortality rates were used to obtain standardized mortality ratios (SMRs), which were stratified for sex, race and age. Internal exposure-dependent trends were obtained for the metrics, peak, average intensity and cumulative exposure, where the RR was set to one in the lowest FA exposure group. SMRs were slightly increased for all cancers, for solid, for respiratory system and for lung cancers (Table 3). For all cancers and solid cancers, the RR was below 1 for peak, average intensity and cumulative exposure in the highest exposure groups. In the highest exposure group, the RR was 0.77, 1.01 and 0.79, respectively, for cancer of the respiratory system. In the highest exposure category, lung cancer was significantly decreased in the peak exposure metric (RR: 0.77), unchanged in the average intensity metric (RR: 1.0) and significantly decreased in the cumulative exposure metric (RR: 0.78). SMRs were significantly decreased for pancreas and breast cancer. No increase was observed for SMRs for solid cancer of the buccal cavity, digestive system, liver, nose and nasal sinuses, larynx, bone, skin, female genital, prostate, bladder, kidney, and brain and central nervous system. The only remarkable effect was the non-significant increase in nasopharyngeal cancer [NPC; SMR (95 % CI) 1.84 (0.84–3.49)]; one misclassified oropharyngeal cancer was included in the SMR calculations, but excluded from the calculation of the RRs. For NPC, the trend was only significant in the peak exposure metric if the non-exposed group was excluded, and the RR was only significantly increased in the average intensity metric in the highest exposure group (Table 4). Overall, this suggests that nasopharyngeal cancer was the only solid cancer associated with FA exposure. With regard to each metric, the middle exposure group had a lower RR than the non-exposed group, suggesting a nonlinear exposure–response relationship with a NOAEC (Table 4).

**Table 4:** Exposure-dependent effect of FA on development of nasopharyngeal cancer in the three formaldehyde exposure metrics in the US National Cancer Institute Cancer Cohort; the reference group was the lowest exposure category in each exposure metric (Beane Freeman et al. 2013)

Peak exposure		Average intensity		Cumulative exposure	
ppm	RR (95 % CI) (*N*)	ppm	RR (95 % CI) (*N*)	ppm × year	RR (95 % CI) (*N*)
0	4.4 (0.3–54) (2)	0	6.8 (0.5–84) (2)	0	1.9 (0.3–12) (2)
**>**0 to <2.0	RR = 1 (1) Reference	0.1–0.4	RR = 1 (1) Reference	>0 to <1.5	RR = 1 (4) Reference
2.0 to <4.0	NA^a^ (0) Apparent NOAEL	0.5–0.9	2.4 (0.15–39) (1) Apparent NOAEL	1.5 to <5.5	0.86 (0.1–7.7) (1) Apparent NOAEL
≥ 4.0	7.7 (0.9–62) (7)	≥1	**12 (1.4–97)** (6)	≥5.5	2.9 (0.6–13) (3)
***P*** **(trend FA groups)** **=** **0.005**		*P* (trend FA groups) = 0.09		*P* (trend FA groups) = 0.06	
*P* (trend FA groups + controls) = 0.10		*P* (trend FA groups + controls) = 0.16		*P* (trend FA groups + controls) = 0.07	

The cohort comprises 25,619 workers. Number of NPC cases is indicated by *N*, and a significant increase is indicated in bold

^a^Not applicable (NA)

The different NCI follow-up studies have been criticized for not adequately addressing heterogeneity between plants (Marsh et al. 2007; McLaughlin and Tarone 2014; Marsh et al. 2014). Furthermore, the statistical evaluations have been criticized for instability of the referent groups with only one NPC case in each of the metrics, for limitations in the trend test, and for the use of non-significant results in the interpretations (Marsh et al. 2014). The critique was further addressed by Marsh et al. (2016) in a re-analysis of the update of the Beane Freeman et al. (2013) study. In the extended 10-year follow-up period, one additional NPC death was observed in the lowest exposure category of highest peak, average intensity and cumulative FA exposure metrics. Repeating the calculations of the Beane Freeman group confirmed that the SMR was increased in the highest peak exposure and the highest average intensity metrics. The Beane Freeman et al. study found no heterogeneity across the 10 plants. In contrast, the re-analysis found a strong heterogeneity between plants in the FA-exposed workers with a SMR of 7.34 (95 % CI 2.69–15.97) in one (the Wallingford) plant, but no increase in the other nine plants with a SMR of 0.82 (0.17–2.41). In the Wallingford plant, the NPC deaths were exposure dependent as, for example, all deaths in the peak exposure metric were in the highest exposure group (≥4 ppm). However, a similar trend was not observed in the other nine plants. In general, the SMRs were higher in the unexposed groups than in the lowest FA exposure groups, used as referent groups (RR = 1) in the Beane Freeman et al. study. As it was considered inappropriate to omit the unexposed groups from the determination of exposure–response relationships, the re-analysis used the unexposed as the referent groups (RR = 1). This resulted in exposure–response analyses showing little or no evidence of associations with peak or average intensity of FA exposure and NPC. The authors concluded that the Beane Freeman et al. analysis was strongly influenced by selection of the referent group, not taking heterogeneity between plants into account and that the re-analysis provided no or little evidence of a persistent association between FA exposure and mortality from NPC.

The risk of nasal cancer, nasal squamous cell carcinoma and NPC was not increased in the Finnish study (Siew et al. 2012). There was no increase in sinonasal and NPC risk (SMR: 0.71) in the British cohort (Coggon et al. 2014). No death from NPC (1.33 expected) or sinonasal cancer (0.95 expected) was observed in the US NIOSH Garment Industry cohort (Meyers et al. 2013). The NCI case–control study on FA effects in the funeral industry also showed a low risk [OR (95 % CI) 0.1 (0.01–1.2)] of NPC (Hauptmann et al. 2009). Nevertheless, using the updated NCI cohort (Beane Freeman et al. 2013) in the risk assessment of portal-of-entry cancers can be considered precautionary, because an increase was not observed in the other comprehensive studies. A nonlinear exposure–response relationship can be suggested, because an increased risk was only seen in the highest exposure groups (Table 4); this is similar to the response for nasal cancer in rats that showed an apparent NOAEC of 2 ppm (Table 2). The new update confirmed that no increase in RR was seen for the peak exposure metric at FA <4 ppm and at the average intensity <1 ppm FA. This was similar to the values from the previous follow-up (Hauptmann et al. 2004) which were used in the WHO (2010) evaluation. Also, the values agree with a NOAEC for SCC in rats at 2 ppm and a lack of nasal histopathological effect at 1 ppm; these values were used in the risk characterization for preventing nasal cancers in humans (WHO 2010).

### Laryngeal cancer

The association between laryngeal cancer and FA exposure was studied in a recent meta-analysis, which included six case–control studies [mRR (95 % CI) 1.15 (0.97–1.36)] and five cohort studies [1.10 (0.84–1.44)] with an overall mRR of 1.13 (0.98–1.31). The authors concluded that the meta-analysis did not support that there is an association between FA exposure and laryngeal cancer (Paget-Bailly et al. 2012). There was no increase in the risk in the three updated occupational cohorts (Table 3).

### Lung cancer in humans

Combined data on lung cancer were constructed from two population-based case–control studies conducted in 1979–1986 and 1996–2002 in Montreal in Canada. A total of 2060 lung cancer cases were compared with 2046 sex- and age-matched controls. Sociodemographic characteristics, lifestyle-habits (including smoking) and complete work history (including work tasks, work conditions and chemical exposures) were obtained from similar interview questions in the two studies. Chemical exposures were evaluated from exposure concentrations (low, medium and high), frequency of exposures (low, medium and high) and years of exposure. Unconditional logistic regression was used to estimate risks [OR (95 % CI)]; confounders taken into account were age, sex, income, number of years in school, ethnicity, smoking, and exposures to occupational lung carcinogens, including asbestos and silica. About 25 % of the cases and the controls were exposed to FA. However, FA exposures were low in the majority of the subjects. For men and women combined, the lung cancer risk for ever- versus never-FA-exposed was not significantly increased [1.06 (0.89–1.27)]. For the group of substantially exposed, the risk was low [0.88 (0.63–1.24)]. Moreover, a long duration of exposure (>20 years) did not increase the risk [0.93 (0.69–1.24)]. Similarly, no increase in risk was observed with age at first exposure, time since first exposure, and maximum intensity of exposure. The authors concluded that FA exposure was not associated with an increased lung cancer risk, which they found was in agreement with majority of studies reported in the scientific literature (Mahboubi et al. 2013). In the NCI cohort, there was no increase in lung cancer risk in the highest exposure category in any of the exposure metrics (Beane Freeman et al. 2013). Neither was an increase observed in the NIOSH garment cohort (Meyers et al. 2013). However, there was an increase in the UK cohort (Coggon et al. 2014), which was not adjusted for smoking. Overall, there is no consistent association between FA exposure and lung cancer (IARC 2006; NRC 2014).

### Cancers at distant sites

In epidemiology, a major focus has been on the association between FA exposure and leukaemia, especially myeloid leukaemia (Hauptmann et al. 2003, 2009; Beane Freeman et al. 2009; Schwilk et al. 2010). Of the human carcinogens identified by IARC, approximately 25 % induce leukaemias or lymphomas. Alkylating agents, benzene, topoisomerase II inhibitors and ionizing radiation induce mainly acute myeloid leukaemia (AML). However, ethylene oxide and 1,3-butadiene are linked primarily with lymphoid cancers (Eastmond et al. 2014). The industries and occupations with the highest occurrence of leukaemia are not those with high exposures to chemicals. Thus, in the US National Occupational Mortality Surveillance System, the highest proportionality mortality ratios (PMRs) for white males (the largest group) are in the industries and occupations: bank/savings and loan/credit agency (PMR: 139–172), advertising/sales manager (PMR: 138–174) and electrical engineers (PMR: 141–192) in different periods between 1985 and 2007 (Robinson et al. 2015).

The NCI Cohort was also followed up until 31 December 2004, for lymphohaematopoietic malignancies (Beane Freeman et al. 2009). Of the 25,619 cohort members, 4359 were classified as never having been exposed to FA. In the follow-up period, 13,951 deaths occurred. The SMRs were calculated using the US mortality rates; they showed no remarkable effect of FA exposure on lymphohaematopoietic malignancies (Table 3). The RR (95 % CI) for death from lymphohaematopoietic malignancies was examined for association with peak exposure, average intensity and cumulative exposure. The group with the lowest FA exposure was selected as the referent group. There was no significant association between cumulative exposure and any of the malignancies. Therefore, results are presented only for the peak exposure and the average intensity metrics (Table 5). The most remarkable increase in RR is for Hodgkin’s lymphoma, apparent from both metrics; this increase was also present in the previous follow-up (Hauptmann et al. 2003), but was not seen in other FA-exposed cohorts as mentioned by the authors. Furthermore, Hodgkin’s lymphoma has not previously been associated with exposures to chemicals (Nielsen et al. 2013; Checkoway et al. 2015); known risk factors are, for example, socioeconomic status, family size and Epstein–Barr virus infection (c.f. Nielsen et al. 2013). In this study, there was no significant association with FA exposures and myeloid leukaemia (Table 3). In the peak exposure metrics, there was a tendency of an exposure-dependent increase in leukaemia (ICD-8 codes: 204–207) as the RRs increased in the four groups from 0.59 (0.25–1.36), 1.0 (referent group), 0.98 (0.60–1.62) to 1.42 (0.92–2.18), respectively, with *P*-trend = 0.02. However, within the three FA groups themselves, the trend was not significant (*p* = 0.12) and the RR in the highest exposure group was not significant. There was no significant relation between FA exposure and RRs of leukaemia in the average intensity metric, where the RR in the highest exposure group (≥1 ppm) was 1.10 (0.68–1.78) and *P*-trend >0.50. This is in contrast to the previous follow-up, where leukaemia was increased in the peak exposure metrics in the middle (2–3.9 ppm) group, RR: 2.04 (1.04–4.01), and the high exposed group (≥4 ppm), RR: 2.46 (1.31–4.62), with a significant trend, and the high myeloid leukaemia group, RR: 3.46 (1.27–9.43), also with a significant trend (Hauptmann et al. 2003). In the new update, the myeloid leukaemia risk was not increased at mean FA exposures below 1 ppm and peak exposures below 4 ppm. To account for the increased risk of Hodgkin’s disease, the mean FA concentration has to be below 0.5 ppm and the peak exposures below 2 ppm even though the disease is not considered to be caused by FA exposure; this is considered to be precautionary as the RR = 1 in the referent groups is roughly in the middle of the 95 % confidence limits of the non-exposed groups.

**Table 5:** Relative risk (RR) of lymphohaematopoietic malignancies in the recent update of the National Cancer Institute Cohort

	Average intensity						Peak exposure				
Disease (ppm)	All	NHL	HL	MM	ML	Disease (ppm)	All	NHL	HL	MM	ML
0	0.99^a^	1.08	0.53 (0.11–2.66)	**2.18 (1.01–4.70)**	0.70 (0.23–2.16)	0	1.07 (0.70–1.62)	1.06	0.67 (0.12–3.60)	**2.74 (1.18–6.37)**	0.82 (0.25–2.67)
>0 to <0.5	1.0 Ref	1.0 Ref	1.0 Ref	1.00 Ref	1.0 Ref	>0 to <2.0	1.0 Ref	1.0 Ref	1.0 Ref	1.0 Ref	1.0 Ref
0.5 to <1.0	1.29	1.20	**3.62 (1.41–9.31)**	1.40	1.21 (0.56–2.62)	2.0 to <4.0	1.17 (0.86–1.59)	1.08	**3.30 (1.04–10.50)**	1.65 (0.76–3.61)	1.30 (0.58–2.92)
≥1.0	1.07	0.71	2.48 (0.84–7.32)	1.49	1.61 (0.76–3.39)	≥4.0	**1.37 (1.03–1.81)**	0.91	**3.96 (1.31–12.02)**	**2.04 (1.01–4.12)**	1.78 (0.87–3.64)
*P* (0 + FA)	>0.5	0.4	**0.03**	>0.50	0.40	*P* (0 + FA)	0.04	>0.50	**0.004**	0.50	0.07
*P* (FA)	>0.5	>0.5	**0.05**	>0.50	0.43	*P* (FA)	0.02	>0.50	**0.01**	0.08	0.13

Beane Freeman et al. Mortality from lymphohaematopoietic malignancies among workers in formaldehyde industries: the National Cancer Institute Cohort. J Natl Cancer Inst 2009; 101: 751–761

*All* Lymphohaematopoietic malignancies (ICD-8: 200–209), *NHL* non-Hodgkin’s lymphoma (ICD-8: 200 & 202), *HL* Hodgkin’s lymphoma (ICD-8: 201), *ML* myeloid leukaemia (ICD-8:205), *MM* multiple myeloma (ICD-8: 203). *Ref* referent group. *P*(0 + FA) is the *P* of trend including all four groups and *P*(FA) is the *P* of trend exclusively in the FA groups

^a^Relative risk [RR (95 % CI)]. The 95 % confidence limits have only been added, where relevant for risk assessment. Significant values are given in bold

The Beane Freeman et al. (2009) study was re-analysed and expanded by Checkoway et al. (2015). The re-analysis subdivided myeloid leukaemia into acute myeloid leukaemia (AML) and chronic myeloid leukaemia (CML); AML is associated with risk factors such as tobacco smoke, benzene exposure, chemotherapy and ionizing radiation, whereas CML is associated with the Philadelphia chromosome, a translocation between chromosome 22 and 9, and with high-dose ionizing radiation. This suggests that AML and CML should be analysed separately. The standardized mortality ratio was 0.80 (95 % CI 0.56–1.14) for AML and 0.96 (0.56–1.67) for CML in the 22,483 FA-exposed workers. A further analysis was conducted in 16,306 workers employed one year or more and restricted to cumulative and peak exposure (which was redefined) as the original study showed no remarkable effect in the average intensity group. Cumulative FA exposures were divided into groups with 0 to <0.5 (referent), 0.5 to <2.5 and ≥2.5 ppm × year and peak exposures into <2 (referent), ≥2 to <4 and ≥4 ppm. In workers exposed for ≥1 year, both cumulative and peak exposures were significantly associated with Hodgkin’s lymphoma (confirming results by Beane Freeman et al. 2009) and all leukaemias combined. In the middle- and high-exposure group, myeloid leukaemia (*N*; hazard ratio (95 % CI) was not increased in the cumulative groups [9; 1.53 (0.54–4.27) and 14; 1.58 (0.59–4.23), respectively, with *P*-trend = 0.39], but was increased in the peak exposure groups [8; 2.49 (1.01–6.16) and 8; 2.03 (0.82–5.03), respectively, with *P*-trend = 0.08]. In the AML groups, risk at cumulative exposure was not increased [6; 1.16 (0.36–3.76) and 10; 1.31 (0.44–3.95), respectively, with *P*-trend = 0.63] and no increase was seen in the peak exposed [5; 1.78 (0.61–5.25) and 5; 1.51 (0.51–4.44), respectively, with *P*-trend = 0.37]. With regard to CML, the cumulative exposed group showed a non-significant increase [2; 2.91 (0.24–35.64) and 4; 3.81 (0.36–40.44), respectively, with *P*-trend = 0.27], similar to the increase seen for the groups with peak exposure [2; 4.83 (0.64–36.42) and 3; 5.32 (0.81–34.90), respectively, with *P*-trend = 0.07]. However, the number of deaths was low. In the full cohort, 13 of the 34 AML deaths were from the group with peak exposures of more than 2 ppm, only four workers had jobs with peaks within the 20 years preceding their death, and only one death occurred within the typical AML window of 2–15 years. Non-Hodgkin’s lymphoma, chronic lymphocytic leukaemia and multiple myeloma showed no increased risk. The authors concluded that the re-analysis does not support that FA causes AML, which they considered the most relevant leukaemia for FA exposure.

A follow-up from the period 1941 through December 2012 was conducted in the British (UK) cohort from six factories comprising 14,008 men (Coggon et al. 2014). In the period, 7378 men had died. A total of 3991 of these men had been highly exposed to FA. In the cohort, the standardized mortality ratio [SMRs (95 % CI)] for all cancers [1.10 (1.06–1.15)], for stomach cancer [1.29 (1.11–1.49)], for rectum cancer [1.23 (1.01–1.49)], for lung cancer [1.26 (1.17–1.35)] was significantly increased based on the national death rate for England and Wales; other values are given in Table 3. No significant increase was seen for cancer in the pharynx, larynx, nose and nasal sinuses, or for the different hematopoietic malignancies. Prostate cancer was significantly decreased [0.80 (0.68–0.94)]. The cohort was stratified for levels of exposure. The exposure in the high-exposure group was more than 2 ppm. A significant increase in SMR in the high-exposure group was observed for all cancers [1.28 (1.20–1.37)], cancer in the oesophagus [1.45 (1.03–1.98)], stomach [1.51 (1.18–1.90)], lungs [1.59 (1.42–1.77)] and the lips [9.98 (1.21–36.04)]; observed/expected: 2/0.2). No increase was seen for non-Hodgkin’s lymphoma [0.90 (0.48–1.55)], multiple myeloma [1.18 (0.57–2.18)], leukaemia [0.82 (0.44–1.41)] and myeloid leukaemia [0.93 (0.40–1.82)]. Exposure in the high-exposure group was further stratified for duration of exposure (<1 year, 1–14 years and ≥15 years). For oesophagus and lung cancer, the SMR was highest in the in the group with the shortest exposure (<1 year), and for stomach and rectum cancer, the SMRs were independent of the length of the exposure period. Additionally, the authors included a nested case–control analysis of cancer in the upper airways, larynx, mouth, pharynx, tongue, and for all leukaemia and myeloid leukaemia. ORs for these cancers were independent of the duration of the exposure. The authors ascribed the increases in risk estimates to non-occupational confounding factors, which may include smoking and socioeconomic factors, and they concluded that the study provided no evidence that FA posed an increased hazard of upper airway cancer or of myeloid leukaemia. It is noted that the study was not able to take smoking and socioeconomic factors into account. Overall, the NOAEC from this study is approximately 2 ppm.

A new follow-up (1960–2008) was conducted of the US National Institute for Occupational Safety and Health (NIOSH) Garment Industry cohort, which is among the largest prospective cohorts (Meyers et al. 2013). The study comprised 11,043 workers. Causes of death were obtained from 99.7 % (3904) of the identified deaths. The year of first exposure was 1970 or earlier for about 77 % of the workers. In the early 1980s, personal FA sampling was performed among 549 employees. The geometric mean FA concentration was 0.15 ppm with a geometric standard deviation of 1.90. No exposure data were available before this time, but FA concentrations are believed to have decreased over time. Recently, NRC (2014) estimated the FA concentration to be about 4 ppm before 1970. Standardized mortality ratios [SMRs (95 % CIs)] and internal comparisons were made using directly standardized rate ratios [SRRs (95 % CIs)] for “duration of exposure”. The SMRs were similar to that of the US population for all cancers, for lymphohaematopoietic cancers (leukaemias, Hodghin disease, non-Hodgkin’s lymphoma, and multiple myeloma), for buccal cavity and pharyngeal cancers, for respiratory cancers, and for brain cancer and other parts of the nervous system (Table 3).

Stratifying SMRs for “year of first exposure” (<1963, 1963–1970, ≥1971) showed no significant increase for lymphohaematopoietic cancers, for trachea, bronchus and lung cancer, and for brain cancer and other parts of the nervous system. Similarly, no significant increase was observed for SMRs for “time since first exposure” (<10, 10–19, ≥20 years). Association with “duration of FA exposures” (<3, 3–9, ≥10 years) was studied with SMRs and SRRs. There was no exposure-dependent increase in risks for lymphohaematopoietic cancers and non-Hodgkin’s lymphoma. The risks increased with the length of the exposures for leukaemia, myeloid leukaemia and acute myeloid leukaemia, but the risks were not statistically significant. For multiple myeloma, the SMR for the exposure groups was 1.2 (0.5–2.3), 2.0 (>1.0–3.6) and 0.6 (0.2–1.6), respectively, and the SRR was 1.00 (reference), 1.2 (0.5–3.3) and 0.3 (0.08 to <1.0), respectively. For trachea, bronchus and lung cancer, the SMR was 1.2 (>1.0–1.5), 1.1 (0.9–1.4) and 0.7 (0.5–0.9), respectively, and the SRR 1.00 (reference), 1.0 (0.8–1.3) and 0.7 (0.5–1.1), respectively. Thus, where the values were statistically significant, they were not associated with the length of the exposure period. However, for individuals with ≥10 years of exposure and ≥20 years since first exposures, leukaemia [23 death, SMR: 1.7 (1.1–2.6)] was significantly increased when multiple causes of death were considered.

Additionally, duration of exposure was studied for leukaemia (36 cases) and myeloid leukaemia (21 cases) using four multivariate Poisson regression models (adjusted for age, year of birth and years since first exposure), where exposures either were untransformed or transformed (log, square root and categorical [<1.6 (reference), 1.6 to <6.5, 6.5 to <16, 16 to <19 and ≥19 years)]. Only the untransformed model for leukaemia and the categorical model for myeloid leukaemia showed a statistically significant trend. However, for leukaemia and myeloid leukaemia, the rate ratios in the categorical model were significantly increased in the fourth category [4.6 (1.3–16) and 6.4 (1.4–32), respectively], but not for the second, third and fifth categories; in the fifth category (exposure > 19 years), no significant increase [2.6 (0.7–10) and 1.7 (0.3–11), respectively] was observed.

The authors concluded that the study showed limited evidence of association between FA exposure and leukaemia, and little evidence of an increased risk of mortality from buccal cavity, pharyngeal (including nasopharyngeal), respiratory and brain cancer, and for Hodgkin’s disease. It is noted that the study lacks appropriate FA exposure assessments and it was not able to take smoking into account. The importance of smoking is not clear; there were sporadically significantly increased values for chronic obstructive lung disease, but no increase in lung cancer.

Also, an Italian cohort with subjects employed in a factory producing laminate plastic, decorative papers and craft papers, using phenolic and melamine resins, has been established (Pira et al. 2014). The major risk was considered to be FA exposure, but FA concentrations were not measured. The cohort comprised 2750 employees from the period 1947 to 31 May 2011, who had been employed for at least 180 days. Data on survival (80.3 %), death (16.6 %, *N* = 457) and emigration (3.1 %) were obtained. Cause of death could not be retrieved for 26 out of 457 (5.7 %) deceased employees. Person-years of observation were 70,933 in the analysis. Expected number of deaths (E) and SMRs were obtained by comparison with the regional death rates. Observed deaths (O) and SMR [O, SMR (95 % CI)] for lymphoma [4; 0.74 (0.20–1.90)], myeloma (O/E = 0/2.3), leukaemia [5; 0.92 (0.30–2.15)] and for all lymphohaematopoietic neoplasms [9; 0.69 (0.31–1.30)] were not increased. Neither was an increased risk of cancer observed for all cancers [149; 0.80 (0.68–0.94)]. The risk was non-significantly increased for oral and pharynx cancer [9; 1.49 (0.68–2.82)] and for bladder cancer [10; 1.51 (0.72–2.77)]. For oesophagus, stomach, colorectal, liver, pancreas, larynx, lung, breast, prostate, kidney, and brain and CNS cancer, the SMRs were below one. The study has a long follow-up period, but a limitation is the lack of quantitative FA exposure data.

Hauptmann et al. (2009) investigated the relationship between mortality and work practices and FA exposure levels among American embalmers in a case–control study. Professionals employed in the American funeral industry who died between 1 January 1960 and 1 January 1986 from lymphohaematopoietic malignancies (*n* = 168), brain tumours (*n* = 48) or nasopharyngeal cancers (*n* = 4) were obtained for 6808 who died in the period and compared with deceased matched controls (*n* = 265) with regard to lifetime work practice. Exposures in the funeral industry were obtained by interviews with next of kin and co-workers, and predictive models were used to estimate levels of formaldehyde exposure. Mean peak concentrations were 8.1–10.5 ppm (model predicted as the maximum 15-minute average intensity ever experienced in connection with embalming for all years) and the average FA intensity was 1.5–1.8 ppm while embalming. Cases were exposed for about 32 years. With one myeloid leukaemia in the referent group, the odds ratio [OR (95 % CI)] for myeloid leukaemia was 11.2 (1.3–95.5) in the ever-embalming versus the never-embalming group. Mortality from myeloid leukaemia increased statistically significantly only with an increasing number of years of embalming (*P* for trend = 0.020) and with an increasing peak FA exposure (*P* for trend = 0.036). Within the exposure groups themselves (duration of years with embalming, number of embalming, cumulative FA exposure, average FA exposure while embalming, 8-h time-weighted average (TWA) FA intensity and peak FA exposure), there were no significant trends in any of the groups (*P* for trend = 0.58–0.97). There was no exposure-dependent effect on monocytic leukaemia, polycythemia vera or myelofibrosis. ORs were roughly about 10 (range 5–15) in exposed groups. To increase stability of the risk estimates, subjects who performed fewer than 500 lifetime embalmings were used as the referent group in a second evaluation. In this analysis, the ORs for myeloid leukaemia were roughly about 3 (range 0.5–3.9) in the “exposed” groups. No true trend tests were available for this evaluation as the authors without explanation used the results from the trend tests from the first (unstable) analysis in this (more stable) analysis. However, ORs were significantly increased for duration of years with embalming at >20–34 years and >34 years, which was 3.2 (1.0–10.1) and 3.9 (1.2–12.5), respectively, with the highest number of embalmings (>3068), 3.0 (1.0–9.2), and at the highest cumulative FA exposure (ppm × hours: >9253), 3.1 (1.0–9.6). These exposures were not related to lymphohaematopoietic malignancies of the lymphoid organs (non-Hodgkin’s lymphoma, multiple myeloma, all lymphoma and Hodgkin’s disease), brain cancer or nasopharyngeal cancer [0.1 (0.01–1.2)].

The validity of the Hauptmann et al. (2009) study has been challenged as it relied only on RR estimates. The expected number of subjects with lymphohaematopoietic malignancies, all myeloid leukaemias and all acute myeloid leukaemias were estimated from the 6808 embalmers (Cole et al. 2010). Comparison between the observed and the estimated number of subjects showed no meaningful elevation. Furthermore, the proportional mortality ratios showed no significant elevation. With regard to risk assessment, it is noted that the study does not provide convincing exposure–response relationships within the different FA exposure metrics, and therefore it cannot be used for setting an indoor air guideline level (Nielsen et al. 2013). Moreover, the potential hazards addressed in this study are not relevant for setting an indoor air guideline level due to the very high exposure levels.

With regard to setting its guideline, the WHO (2010) used the Hauptmann et al. (2003) study as the key study for risk evaluation of lymphohaematopoietic malignancies. The new update (Beane Freeman et al. 2009) weakens the association between FA exposure and leukaemia and myeloid leukaemia, indicating that the WHO (2010) guideline is based on conservative estimates. Additionally, the guideline is set below levels associated with increase in Hodgkin’s lymphoma, although this disease has neither been consistently associated with FA exposures nor with exposure to other chemicals.

## Evaluation of carcinogenic risk and the WHO (2010) indoor air quality guideline

The two critical effects of FA are nasal cancer and irritation of eyes and upper airways (sensory irritation), where sensory irritation had the lowest NOAEC and is the basis of the WHO IAQG at 0.1 mg/m^3^ (0.08 ppm), applying to each 30-min period during each day lifelong. It is noted that the NOEAC for sensory irritation is 0.5 ppm and thus an assessment factor of 6 is used when setting the IAQG, which is a conservative approach. Moreover, this guideline value is considered to prevent all portal-of-entry effects, including nasal cancer, and potential systemic cancers. Even though the potential systemic cancer effects are considered not to be relevant with regard to setting an indoor guideline, all endpoints were evaluated to assure that the guideline level was below effect levels to ascertain a contradiction-free guideline (WHO 2010; Nielsen and Wolkoff 2010; Nielsen et al. 2013).

Cancer risk assessment of indoor air exposures to FA has been carried out based on two different approaches. One approach is linear extrapolation (e.g. Sarigiannis et al. 2011; Huang et al. 2013; Du et al. 2014; Rovira et al. 2016), which provides conservative estimates (e.g. Nielsen and Wolkoff 2010; Sarigiannis et al. 2011; Du et al. 2014). As FA is an endogenous metabolite, at a certain low exogenous exposure level, the cellular FA level will be dominated by the endogenously (naturally) generated FA and exogenous FA contribution will be low or negligible (Andersen et al. 2010; Swenberg et al. 2013). Based on levels of endogenous and exogenous FA–DNA adducts, it was shown that the linear extrapolation approach greatly overestimates the cancer risk (Starr and Swenberg 2013, 2016).

In 2010, the WHO launched a threshold approach for establishing an IAQG for FA. Analysis of cancer exposure–response relationships in experimental animal (e.g. Table 2) and human epidemiological studies showed that the relationships were nonlinear (WHO 2010). Additionally, the mucosal metabolism would prevent systemic access of FA when airborne concentrations were below a few ppm FA.

With regard to experimental animal species, rats were the most sensitive to developing nasal cancer due to lifelong exposure with a LOAEC of 6 ppm and an apparent NOAEC of 2 ppm (Table 2). Nasal cancer was driven by cytotoxicity induced cell proliferation (Conolly et al. 2003, 2004). This may cause DNA transcription before DNA lesions were repaired and thereby allow the DNA damages to be fixed as mutations, which in turn may cause cancer. The NOAEC for increased cell proliferation was >2 ppm (Monticello et al. 1996; Andersen et al. 2010). The histopathological NOAEC (1 ppm) for damage of the nasal epithelium was lower than the NOAEL for cell proliferation and for nasal cancer, for which reason the histopathological NOAEC was accepted as the point of departure for the cancer risk assessment. Also, it was accepted that the histopathological NOAEC was independent of the duration of the daily exposure period and therefore NOAEC was considered to be a full day of exposure. Furthermore, rats were considered more sensitive than humans. Due to the local airway effect, the 1 ppm level was divided by an interspecies assessment factor of 3 (it is noted that rats are a sensitive species) and an assessment factor of 2 for the limit variations within the human population [deposition in the upper airways is similar in children and adults, and the variation in the population is about 1.6-fold (Garcia et al. 2009)], resulting in a guideline value of 0.17 ppm (0.2 mg/m^3^) for protection against nasal cancer. It is noted that the assessment factors are highly conservative. This approach was backed up by a comprehensive biologically motivated computational modelling method, predicting that the 80-year lifetime additional risk is ≤10^−6^ at 0.2 ppm (0.246 mg/m^3^) for non-smokers (Conolly et al. 2004). Preventing nasal cancer was considered to prevent systemic cancers, including leukaemia.

The credibility of the histopathological endpoint in the risk assessment of cancer is further supported by recent animal studies. For example, the toxicokinetic studies by Andersen et al. (2010) showed that the local airway epithelial cell FA level was not markedly affected by FA exposures from 0.7 to 2 ppm, and at 1 ppm, the epithelial FA concentration was dominated by the endogenous (natural) FA concentration. At these concentrations, the exogenous FA–DNA adducts were lower than the endogenous adducts in the rat nasal tissue (Lu et al. 2010, 2011; Moeller et al. 2011; Edrissi et al. 2013;Yu et al. 2015). Moreover, the recent studies have confirmed experimentally that apart from the portal of entry no absorption occurs in rats at exposures in the range 0.7–15 ppm (Lu et al. 2010, 2011; Edrissi et al. 2013; Kleinnijenhuis et al. 2013; Yu et al. 2015) and in monkeys up 6 ppm (Moeller et al. 2011). Overall, this supports the credibility of the selected point of departure by the WHO for low-level portal-of-entry effects, which if prevented also prevent systemic effects.

The WHO (2010) IAQG was also supported by epidemiological studies. In the previous update of the NCI cohort, nasal cancer occurrence had a nonlinear exposure–response relationship where no increased risk was observed below 1 ppm average intensity and below 4 ppm peak exposures (Hauptmann et al. 2004); the exposure–response relationship was nonlinear. The Hauptmann et al. (2004) study was used as a key study by the WHO. The cohort was recently updated (Beane Freeman et al. 2013) with similar results. However, no increase in nasal cancer was observed in later comprehensive studies and updates (Siew et al. 2012; Meyers et al. 2013; Coggon et al. 2014) and a case–control study (Hauptmann et al. 2009). In the latest update of the NCI cohort (Beane Freeman et al. 2009), myeloid leukaemia risk was not increased at mean FA exposures below 1 ppm and peak exposures below 4 ppm and thus shows that preventing nasal cancer also prevents leukaemia. Remarkably, the previous follow-up study (Hauptmann et al. 2003) and the most recent follow-up (Beane Freeman et al. 2009) of the NCI cohort both showed an increase in Hodgkin’s lymphoma at the average intensity ≥0.5 ppm and peak exposures ≥2 ppm. Hodgkin’s lymphoma has not previously been associated with exposures to chemicals (Nielsen et al. 2013; Checkoway et al. 2015); known risk factors are, for example, socioeconomic status, family size and Epstein–Barr virus infection (c.f. Nielsen et al. 2013). Therefore, it is considered sufficient that the guideline is below these levels.

On the whole, the nonlinear exposure–response relationships, the epidemiological effects at levels much higher than the WHO IAQG and the lack of consistency across studies indicate that the WHO IAQG is highly precautionary.
